# Navigating the path to a biomedical science career

**DOI:** 10.1371/journal.pone.0203783

**Published:** 2018-09-07

**Authors:** Andrea M. Zimmerman

**Affiliations:** Department of Education, Northeastern University, Boston, Massachusetts, United States of America; Charles P. Darby Children’s Research Institute, UNITED STATES

## Abstract

The number of biomedical PhD scientists undergoing training and graduating far exceeds the number of academic faculty positions and academic research jobs. This trend compels biomedical PhD scientists to increasingly seek career paths outside of academia. Prior studies have used quantitative methodology to determine trends and outcomes of single factors contributing to this shift, but there is a literature gap in studies considering multiple factors and in qualitative work focusing on biomedical PhD scientists’ experiences and their processes of career navigation. This paper draws on a social cognitive career theory (SCCT) framework and incorporates case study data from a southeastern Tier 1 research university to explore a nexus of factors influencing PhD scientists’ employment sector preferences and job search processes. It simultaneously concludes that relationships with faculty, particularly the mentor advisor, are essential to the opportunities available to these professionals and to the career paths they choose.

## Introduction

Academia has traditionally been the preferred career for PhD scientists, but available tenure-track faculty positions are increasingly scarce in comparison to the number of highly-qualified candidates, creating a grim outlook for graduate students and academic institutions [1]. Overemphasis of the academic track creates tension through stigmatization and devaluation of careers outside of academia by advisors, institutions, and funding sources [2], as many PhD scientists choose or are obligated to enter non-academic, or non-traditional, careers. In this work, non-academic and non-traditional are used interchangeably and used to mean any position that is not a tenure-track academic research position. Individuals are less likely to go into biomedical research without a reasonable chance of finding a respectable job in their field after graduation [3–4].

Most current research has focused on single factors affecting career choice: oversupply and insufficient demand for PhD scientists in academia [4–11], the rising median age of professional achievements and prolonged training periods [4–6, 12–13], and internationalization [3, 8, 14–17]. Quantitative studies have reported on trends and outcomes of biomedical PhD scientists pursuing employment by using the traditional academic pathway as the definition of success [16, 18–25]. A review of the literature revealed hardly any qualitative research, and most publications were editorials and opinion pieces rather than empirical studies. The case analysis presented here contributes to understanding the complex employment dilemmas biomedical PhD scientists face, and explores influential factors determining the options they have and decisions they make as they negotiate their careers.

### The availability of academic faculty positions

Academia is no longer the primary career for biomedical scientists who have shifted over time to consider academia as a secondary employment type [15, 26]. From approximately 1965 to 2017, the proportion of science PhD holders who obtained tenured academic positions has dropped steadily; more PhD degrees were awarded than the number of positions available [4, 6, 8–10]. Between 2005 and 2009, 100,000 doctoral degrees were awarded, but only 16,000 new professorships were created [27].

Tenured faculty positions have been traditionally assumed to be the motivating factor for seeking a biomedical science PhD; however, attaining these positions becomes more and more difficult as universities replace tenured faculty with non-tenured staff [28]. Many biomedical PhD scientists now work outside of academia. In a 2006 survey of scholars who had completed PhDs 5–6 years prior, 43% were in non-academic careers, 17% had non-tenure track positions, 10% worked part-time (or were out of the labor force), and 17% were still postdocs [1].

Many biomedical PhD holders are turning to work in industry, though the number of available industry positions has not consistently compensated for the decrease in academic tenure track positions, as graduate schools have not limited enrollment accordingly or offered training needed for alternative careers [23]. Supply still overwhelms the combined academic-industry demand. Even though few PhD holders end up unemployed, often the years spent on training are not justified for the jobs they actually acquire [8].

### The economic market

Applications and enrollment in graduate school have remained steady despite the proportional decrease in available positions, causing the employment market for biomedical professionals to contradict traditional economic assumptions. As financial resources from the U.S. federal government—the largest investor in higher education—shrink, institutions have been simultaneously pressed to maintain or boost academic quality and integrity while reducing reduce spending. When fewer trainees stay in academic research, it simultaneously and negatively affects principal investigators’ grant funding, number of papers published, and reduces governmental funding support of graduate programs.

The National Institutes of Health (NIH) is the main U.S. organization funding graduate students and academic research in biomedical science [28]. When its budget doubled in 2003 to fund bioterrorism research, universities could not sustain all of the additional jobs created [7]. Indeed, “biomedical research funding is both erratic and subject to positive-feedback loops that together drive [the] system ineluctably toward damaging instability” [11]. Markets experience expansions and contractions, but biomedical science does not respond, or responds sluggishly, to classic market forces because infrastructure cannot change as rapidly as funding levels [5].

### Delayed scientific independence

Increased standards for publishing papers and receiving grants have contributed to the amount of time biomedical scientists must remain in training, causing careers to begin later. Grant applications now require more “preliminary” data—a project must be nearly complete before it is proposed [12–13]. Researchers [4, 6] reported that on average, scientists received their first NIH grant at age 42. The best science faculty members train far more scientists than are needed to replace themselves, generating a supply much greater than the relevant positions in academia can absorb. Overproduction may be due to demand for research assistants and postdocs rather than a demand for scientists in permanent roles [29]. Reform has been limited, primarily because supply continues to benefit the most established and influential scientists and undoubtedly produces great science [5].

### Individual perceptions and hurdles

The availability of positions affects students on the track to become academic faculty members, but the perception about types of career options also has a major impact on whether or not a student will pursue academic science. Desires and intention drive students’ preferences before and entering graduate school, but perceptions and views of each sector begin altering desires and intentions during training.

#### Desires and intention

When students begin graduate school, most consider the faculty research path to be among the most desirable career options for them, but many felt forced to leave academia after graduating [16, 24, 29–31]. Deciding a direction at the beginning of a PhD program, when information about career options is limited, may subject students to a lock-in phenomenon [16]. From a sample of 3,800 doctoral STEM (science, technology, engineering, math) respondents and data from The Survey of Doctoral Recipients/National Science Foundation, career preferences were 41% non-academic, 36% academic research, 19% academic teaching, and 4% a combination [29].

Scholars of trends in biomedical academics have studied the supply and demand problem in depth, but few have closely examined how career preferences adjust over the course of education and the role advisors play in influencing this process [24]. Career preferences often shift during the third year of graduate school [1], especially as students begin to struggle with a different understanding of the academy than they had originally anticipated [32].

#### Perceptions and pressures of academic life

Many factors lead students and some new faculty members away from their initial planned career paths. Some are professional: disappointment in academy and research, low availability of jobs, loss of interest in basic research or in specific sub-areas, and multiple and possibly conflicting professional responsibilities. Others are personal: isolation, lack of collegiality, stress, work-life balance, lack of institutional family-friendliness, household income, availability of child care and health insurance, and competition [1, 17, 29, 32–35]. While academic faculty salaries are low, the main concern is that they have not risen with inflation [36–37], and teaching loads have increased while research budgets have decreased. Being asked to do more with less is only offset by the intrinsic rewards of academic work.

#### “Nontraditional” Versus “Alternative” Careers

Though many postdoctoral fellows prefer academic research careers, 64% are interested in careers outside academia; however, 70% of advisors do not discuss nontraditional career paths, and about 20% are not supportive of non-academic careers [38]. The bias may be unintentional, but it still exists. Some students report that nontraditional careers in industry, and teaching primary and secondary grades, are explicitly discouraged [24].

Funding agencies, faculty, and graduate programs continue to use the traditional academic pathway as the definition of success. Programs are often evaluated on the number of alumni in academia, particularly whether an alumnus holds a principal investigator-level academic faculty position [1]. “Nontraditional” career paths are commonly referred to as “alternative,” a moniker with negative connotations for viable career options. Scholars now prefer “career choices” [6], “career paths,” or “career options” in an attempt to shift the descriptions of sectors and career paths to begin to change the stigma of non-academic careers [1].

### Equilibrium efforts to move from pipeline to pathway

Scholars have increasingly advocated for the development of curricula to prepare PhD holders for more diverse career paths [1, 14–15, 32, 39–40]. Wendler et al. [28] called the trend in trainees pursuing paths outside of academia a “leakage from the pipeline”—and [17] suggested career prospects follow a pathway more than a linear approach. Researchers [5, 41] recommended that mentors and institutions should help transition young scientists into a broader range of careers that would benefit from their specialized education and abilities; they also suggested that institutions should gradually reduce the number of PhD trainees to better match available opportunities.

Indeed, given that job opportunities for U.S. scientists largely exist outside of academic research [40] and the line between academic and industry or entrepreneurship is progressively blurring [42–43], scholars have argued that graduate education paths must be increasingly designed to encourage the pursuit of these options. In this shift, the advisor plays a critical role, by identifying, nurturing, and moving the next generation past what they could accomplish [44].

Guiding students to non-academic careers is challenging and may be opposed by current faculty members, institutions, and funding sources. However, when the number of academic positions is limited and shrinking, new pathways must be encouraged and resources provided to help students strategically prepare for non-academic careers.

The case study described in this work has identified that the current system of training biomedical scientists is inadequate for the career opportunities available post-training, and looks at the factors, such as personality, environment, experiences, perceptions, etc., that influence the job search process. Faculty mentors are the most influential factor navigating the career search and path.

## Methods

The exploratory case study described in this work contextualized the experiences of biomedical PhD scientists navigating career paths. The case study approach allows for a detailed and in-depth look into data over time within a context through multiple sources of information and data collection and supports the emergence of themes and patterns through inductive reasoning to explore a contemporary phenomenon [45]. Each data source type includes its own sampling, data collection, and analysis strategies [46], particularly to provide depth, context, and validity.

Though the study’s intent was to learn about non-academic career paths, data were also collected about and from academics for contextualization and cross-analysis. This qualitative case study used a descriptive survey, a focus group with current trainees, interviews with three types of stakeholders (trainees, administrators, and faculty), and document review to generate data. Data were organized and analyzed after a multi-step coding process using Saldaña [47] as a guide. The following is a brief description of the methods used in this study. For a more complete description of the methods used in the larger research study, refer to the S1 Appendix.

This submission is original work of the author, who has no financial or other conflicts of interest. All standards for human subject research were followed during the course of the investigation, and approval was received by the Northeastern University IRB (IRB #CPS16-04- 10). Site permission was granted at the research site described below, and participants provided written consent to participate in the study. The anonymized data that support the findings of this study are openly available in Open Science Framework (OSF) at https://doi.org/10.17605/OSF.IO/25MFU.

### Theoretical framework

**Fig 1:**
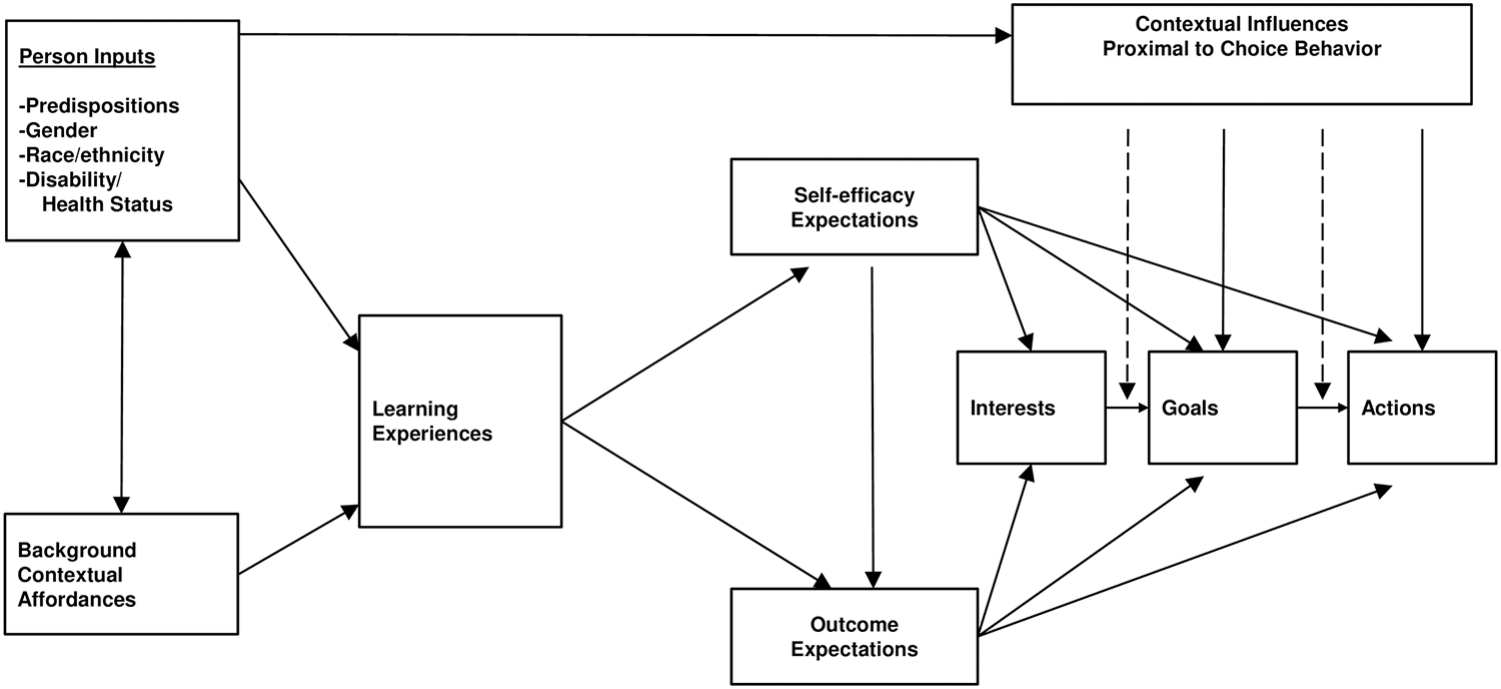
Social cognitive career theory model. This model demonstrates the relationship between personal, environmental, and experiential factors and their influence on goals, expectations, and actions. Reprinted with permission.

This study used the Social Cognitive Career Theory (SCCT) as the theoretical framework to examine how various factors influence career choices (Fig 1) [48]. The theory and model assisted in explaining and predicting processes through which students develop vocational and academic interests, make choices regarding academic and professional pursuits, and attain specific levels of work and academic performance.

SCCT is more comprehensive than previous theories such as social learning theory, using cognitive mediators through which learning experience guides career behavior; it incorporates personal and behavioral variables, contextual factors influencing career outcomes, and the exercise of personal agency in career development. Major components of this theory include identifying occupation options, analyzing actual and perceived barriers, and modifying self-efficacy beliefs, all which were incorporated into this study.

The first set of factors examined as part of this research included the individual’s characteristics, experience, background, and interests upon entering graduate school. The second set involved learning and performance in graduate school; skills acquired; the career search; and interactions with mentors, faculty, and other students and researchers. The final set addressed the career path and necessary skills and abilities that should have been acquired in graduate school and postdoctoral training to aid in career development and the transition to work in or out of academia. I consider these factors in relation to the career path navigated.

### Research site

The specific NIH-supported training program, within a single institution, and sample characteristics for this study were selected for multiple reasons. First, the focus of NIH training grants is to train biomedical scientists for academic research; however, many do not intend to do so or do not enter academic positions. The training program examined at the research site is one of the largest and longest running in the United States, with trainees spanning many degree areas and most departments. This program also supports and trains both graduate students and postdoctoral fellows, which is not typical of many NIH training grants. At the time this study was conducted in 2016, the program was supporting 15 trainees per year. Some trainees were also supported for multiple years. The training program has existed for more than 40 years, so its longevity, the combination of pre- and postdoctoral trainees, and the number of trainees who had been supported by the program together created a large sampling frame.

### Sample characteristics and procedure

The three groups of participants in this case study were *All Trainees*, *Faculty*, and *Administrators*. All Trainees were further subdivided into current trainees (*In Training*) and former trainees (*Trained*). Individual interviews were the primary method of data collection and were supplemented by the survey, document collection, and a focus group. A topical focus group allowed the researcher to bring together people who had experienced similar training to allow the nuances of backgrounds, perceptions, and experiences to come through responding to the researcher and other focus group participants, which indeed differed from individual interviews [49].

#### All trainees

All past and current graduate students and postdoctoral fellows who had been supported on this NIH training grant, and who had current contact information available at the time the study was conducted (*All Trainees*), were contacted to participate in a comprehensive online survey with 19 closed- and open-ended questions.

Of the 306 trainees who had been part of the training program, 88 could not be found or did not have current email information, at least two former trainees were deceased, and one was stationed in a remote international location without access to the Internet. There were 214 potential survey participants, and 98 completed the survey. Though all potential survey participants may not have received the invitation, the survey response rate was at least 45.79%.

Those individuals who self-selected into the second level of the study were separated into two groups: those who were currently *In Training* and those who had completed training (*Trained*). More than one-third (37) of the survey respondents volunteered for the next phase of the study. However, only 34 were considered for the sample, as three did not leave name or contact information in the anonymous survey.

The *In Training* group included those who were being or had been supported by the NIH training grant and who were still in training at the institution. Six *In Training* survey participants volunteered to participate in a focus group; two were unable to participate due to travel and clinic schedules. For document review, *In Training* participants submitted lecture notes from five career development seminars, a research statement and career objectives for application to graduate school, and a personal statement for application to the training program.

The *Trained* group included those who had completed training. The majority of data was derived from qualitative, semi-structured interviews with the *Trained* group of PhD scientists. The structure and technique for conducting interviews was guided by Seidman [50]. The volunteers for interviews were fairly representative of the training program makeup (see Table 1) so the researcher did not oversample any particular group. Several volunteers had experience in multiple sectors and were selected, even if they were currently working in the academic sector, because of their non-typical career path.

**Table 1:** Demographics of *In Training* and *Trained* participants.

Trainee	Years in Training	Pre or Post-doc	Sex	Current Career Sector	Prior Career Sectors	Career Sector Interest Prior to Graduate School
1	Current	Pre	F	Not applicable	Not applicable	Academia, Healthcare
2	Current	Pre	M	Not applicable	Not applicable	Industry
3	Current	Pre	F	Not applicable	Not applicable	Healthcare, Government
4	Current	Post	F	Not applicable	Not applicable	Academia
5	1991–1995	Post	M	Academia	Industry	Academia, Industry
6	1993–1998	Pre	M	Non-research	Industry	Academia, Healthcare
7	1990–1998	Pre	F	Government	Academia	Academia
8	1998–2003	Pre	F	Non-research	Academia, Industry	Industry
9	1998–2003	Pre	M	Academia (non-traditional)	Academia, Government, Industry	Healthcare, Industry
10	1981–1988	Pre	M	Industry	Academia, Industry	Academia
11	1994–1998	Pre	F	Government	Government, Industry	Industry
12	1979–1985	Pre	M	Industry	Academia, Industry	Academia

#### Faculty

Qualitative interviews with *Faculty* comprised the next phase of the study. These interviews were also were semi-structured, flexible, and included mostly open-ended questions.

Faculty mentors who had trained graduate students and postdoctoral fellows supported by the main NIH training grant, or though other grants or means, constituted the *Faculty* group. There were 46 faculty mentors across 13 departments or divisions with a wide range of experience contacted to participate in the study. Four faculty members volunteered to participate in an interview (see Table 2). For the document review, one faculty member submitted a personal statement for a grant and a career development presentation.

**Table 2:** Demographics of *Faculty* participants.

Faculty	Rank	Sex
1	Professor	F
2	Assistant Professor	M
3	Associate Professor	F
4	Associate Professor	M

#### Administrators

Qualitative interviews with *Administrators* comprised the final phase of the study. These interviews were also were semi-structured, flexible, and included mostly open-ended questions.

Thirty associated program administrators at the program management and director levels of departments, other NIH training grants, the Office of Postdoctoral Affairs, and the Graduate Programs Office (*Administrators*) were contacted for an interview to learn about the training process and opportunities available for graduate students and postdoctoral fellows outside of the research laboratory. Two program administrators elected to participate in an interview (see Table 3). One administrator submitted one new career development course description for document review.

**Table 3:** Demographics of *Administrators* participants.

Administrator	Time in Position	Sex
1	2 years	F
2	More than 10 years	F

### Data analysis

#### Close-ended survey questions

The close-ended survey data allowed the researcher to calculate descriptive statistics, look for outliers, and search for patterns and key words or phrases. Though this study includes some quantitative data, it is predominately a qualitative study and was analyzed as such.

#### Open-ended survey questions, focus group, interviews, and documents

Responses from the focus groups and interviews were transcribed verbatim. The transcripts were provided to the participants for review as a method of member-checking. Finally, the transcripts were uploaded into MAXQDA to code along with the open-ended survey responses and collected documents using Saldaña [47] as a guide generating general thematic codes and then iterative patterns. Finally, provisional coding was used with categories and themes from the literature review and the SCCT.

## Results

The findings are categorized into themes, which are organized according to social cognitive career theory (SCCT). Factors influencing the job search process for these trainees included: diversity, personality, training environment, perceptions, influential people, goals, and interests. Perceptions and influential people were particularly significant in guiding the career path for these scientists.

### The process and outcome of the job search

**Fig 2:**
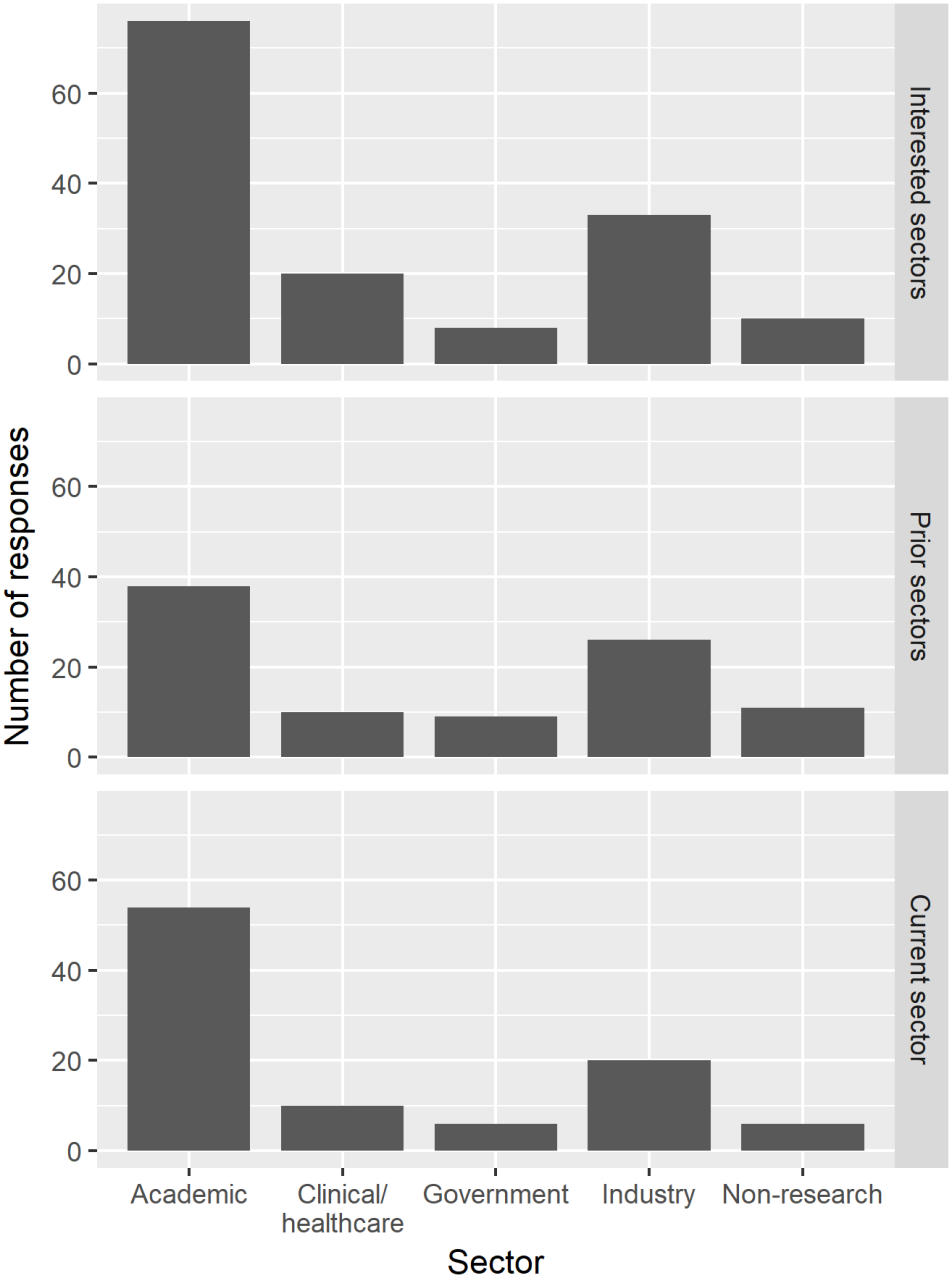
Career sectors. The interested sectors panel displays the career sectors trainees intended to pursue upon entering training at the research site. Note that this counts responses, not respondents, as each respondent could select more than one sector. The prior sectors panel illustrates survey respondents’ prior career sectors. Note that some respondents have worked in multiple sectors, while most current trainees have not been employed in a sector. The current sector panel shows the distribution of survey respondents’ career sectors.

These biomedical PhD scholars asserted that the job search process was easier when they had strong networks and were open to multiple sectors. Fig 2 shows the current and prior career sectors for survey respondents. Both show an over-response for academic careers, as current trainees at the research site and those in postdoctoral training at other institutions selected academics as career sectors, even though none had technically entered a career path. Almost 75% of survey respondents said they felt relatively (answering 7 or above on a 10 point Likert scale) prepared for a career, but responses were much more varied for the degree to which respondents felt prepared for the career search.

### Intended and desired career

Personal interests, goals, and motivations certainly guided PhD biomedical scientists’ interest in science, their area of focus, and their career paths. Self-efficacy and interests are reciprocal influences.

Faculty expressed that they thought students had a good idea of their interests and career path when they began graduate school. In her experience, Faculty 3 found about 50 percent of the students showed interest in academic careers and 50 percent sought non-academic careers. The students’ personal statements, however, commonly discussed getting a PhD because of hopes of obtaining a career in academia, which was the only sector most of the graduate students knew of as a career option when they entered school. Indeed, the survey showed that trainees’ non-exclusive career sector interests prior to graduate school were: 77.6% (76 out of 98 respondents) academic, 33.7% (33) industry, 20.4% (20) clinical/healthcare, 8.2% (8) government, and 10.2% (10) non-research (Fig 2). A couple of the trainees who had experiences outside of academia in or after undergraduate education, such as internships or taking a few years off to work, were more open to and interested in industry and other environments.

Only a few interviewees said the career paths they had taken were their intended or desired career paths. Unfortunately, some trainees who went into their intended careers outside of academia were viewed as failures by faculty and funding programs. This stigma by faculty and funding programs hindered the trainees’ navigation of their intended career paths. Administrator 1 provided an example of a common situation, according to trainees, faculty, and administrators:

One of the post-docs I know is in a position like that. It’s 80 percent teaching, 20 percent research. You are a success if you do 80 percent research, 20 percent teaching. She’s looked upon now as a failure on the training grant. I think it’s unfair. It’s what she wanted to do from the beginning teach–so I just think that it’s …people sometimes go through all this trouble. They love teaching, and they love the subject matter, and they want to be in the forefront when things change or when science changes or whatever. They want to be part of that, the teaching process. I think it was kind of wrong how it was looked upon that she settled and too bad that we put so many resources into her, and this is what we’re going to get out of her.

Desired career paths changed during the training period, and trainees seemed to be more open to all career sectors than faculty believed them to be.

### Influential interactions

Beyond personality, interests, desires, environmental factors, and aptitude, current and past biomedical science graduate students and postdoctoral fellows named people who had influenced their academic focus or career path. Several were influenced by family, friends, peers, or colleagues; the majority of trainees, however, mentioned that their strongest influencers were their mentors and advisors. Faculty encounters during training were essential to success in the training phase, the career search, and career skill development.

Half of the trainees struggled even to think of an influence other than an advisor or mentor. Trainee 4 regarded her undergraduate mentor as “fantastic” and as the person who “really motivated [her] to pursue science, like consider science as an actual career.” Although his family included scientists and medical professionals, and he knew he would go into some area of science, Trainee 12 considered his mentor as the one who drove the career path he chose, after training and “even today.” Faculty 2’s mentor influenced his career path by encouraging trainees to remember funding “goes through cycles, [they have] had this before, this is going to get better, this is not something [they] can worry about too much if [they] want to be a scientist.” The positive attitude expressed by the mentor, who refrained from complaining about writing or not receiving grants, made a big difference in not discouraging the student from seeking an academic career.

### Positive faculty interactions

Interactions with faculty mentors and other faculty members also impacted biomedical PhD scientists’ training; it shaped their navigation of their career paths. Mentors had a tremendous effect on the process and support of trainees.

#### Interactions with mentors were greatly influential

**Fig 3:**
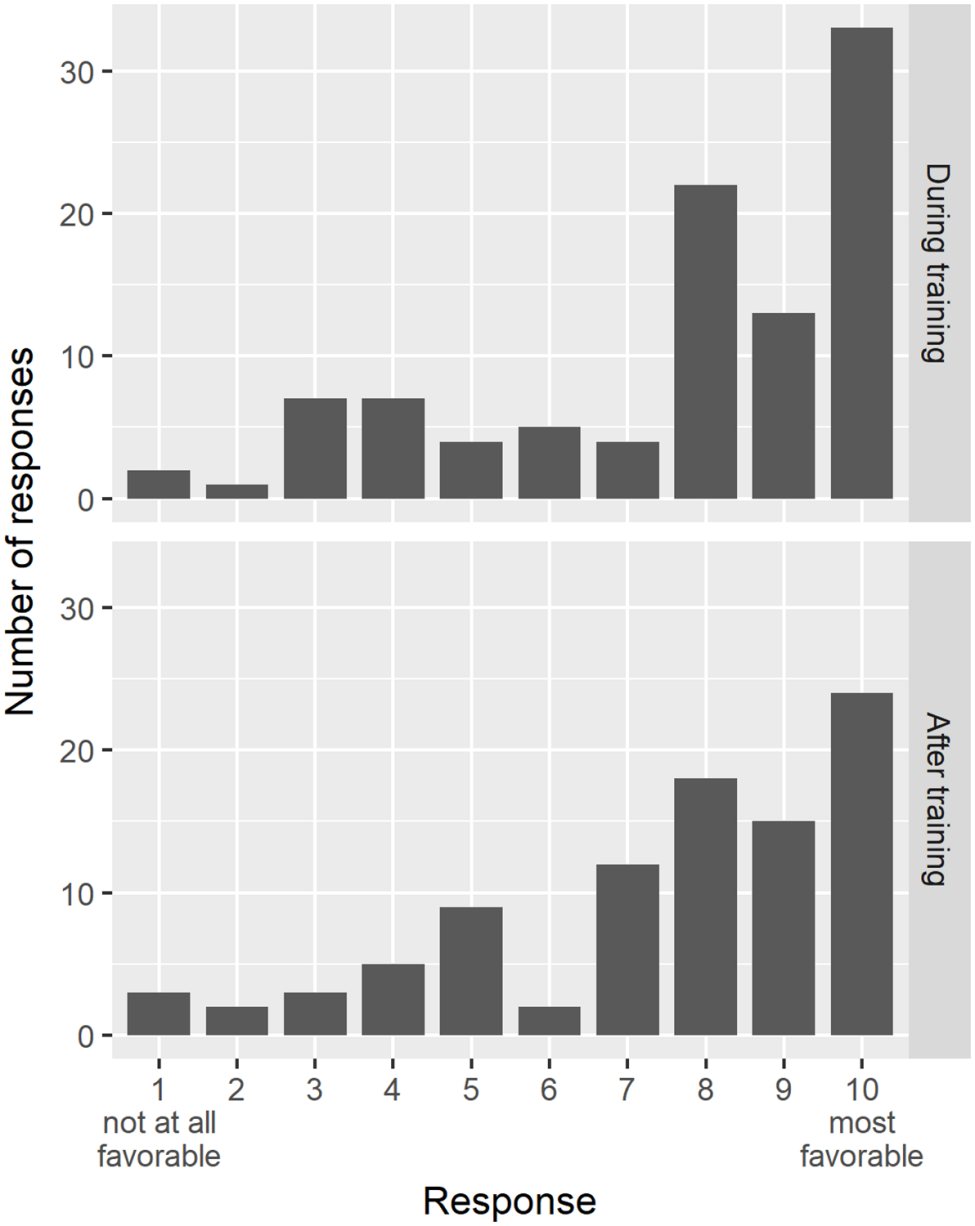
Mentor interactions. The during training panel illustrates how favorable survey respondents’ found their interactions with the mentor during training. The after training panel illustrates how favorable survey respondents’ found their interactions with the mentor after training.

Interactions with mentors was a key aspect of training and support given to graduate students and postdoctoral fellows. Those who responded to the survey described the extent to which their interactions with their mentors were favorable during and after training. During training, interactions were quite favorable, with 69.4% (68 of 98 respondents) of respondents selecting eight or greater on a 10-point scale; Interactions were more positive during training than after (Fig 3). The document review provided the following list of traits of a good mentor: “accessibility, empathy, open-mindedness, consistency, patience, honesty, savvy, responsive.”

Trainee 10 stated that he appreciated the way his mentor interacted with him and the other lab members, guiding them toward a solid career path. He described: “It was very much an individual thing. You were either working for somebody who was interested in helping you with your career or you weren’t.”

#### Engagement with secondary faculty members provided additional training and support

Engagement with faculty other than one’s own mentor was generally positive. The document review showed that trainees tended to underutilize their committee members. Trainee 1 said she was attracted to a specific research site over her other choices because secondary faculty reached out to her individually after her visit.

Trainee 7 said multiple committees of faculty members exposed her to “interactions between the conversations between medical science research and basic science research, and where the money comes from, and where the money doesn’t come from, and technical aspects like that.” The institution she was describing in that case had a pre-doctoral committee, which advised trainees while they were involved in their research. The thesis committee, however, judged the written and presented thesis in its more complete form. Committee members from the two groups came from different backgrounds or worked at different institutions. Trainee 10 said he saw the benefit of faculty engagement and thesis committees, and that he thought the only trainees that had “a rough time in their dissertation defense” were those with committee members who were too narrowly focused and did not appreciate the implications of the trainee’s work.

Administrator 2 observed how seriously the director of the training program took faculty engagement. For example, this particular director met with the program faculty, or preceptors, annually to go over expectations, goals, activities, etc. for the year. He commonly told the faculty members that, if they did not or could not meet the expectations outlined in the training structure, they would be removed from the list of participants. The program faculty were also vetted by a program steering committee that reviewed the preceptor list; individuals who were evaluated as not adding value to the training community were dismissed. Some faculty members appeared to consider training activities as simply checking an attendance roster; others did not add value to the program or commit to the other activities that could have benefitted the trainees, such as attending student presentations to provide feedback or providing a career development seminar. When the faculty were not fully engaged in the program, they were seen as using the program as a funding source to support their student or post-doctoral fellow. This demonstrated the importance of faculty engagement for training programs.

### Negative experiences with mentors

Several trainees had negative experiences with mentors which were most often a result of deficiencies in personnel management, guidance and direction, and engagement. Personnel management was discussed at length during the focus group. Though personnel management is the responsibility of the faculty mentor, several trainees were left to manage personnel issues and to deal with conflict, which they said hindered productivity. Some conflicts were interpersonal and not related to science or research, but they still negatively impacted the trainees’ desire to pursue an academic career.

For example, at the beginning of her graduate studies, Trainee 1 became the first author on one article and the second author on another, and she was asked to provide review consultation within the first 18 months of her studies, which represented a high level of performance for a graduate student. The success seemed to create a competitive edge with at least one of her peers. She stated that this student began harassing her and behaving aggressively, to the extent of stealing equipment from her and committing other actions that hindered her projects and progress. The situation was made known to her co-mentors, but no action was taken for a year and a half. The trainee became discouraged at the lack of recourse, and said she felt it was not handled properly or at all. The faculty’s ignorance of the issue enabled the other student to continue interfering with Trainee 1 personally and professionally, making the lab a difficult place for her to continue her scientific work, seriously decreasing her productivity. She explained:

I expected [graduate school] to be hard. I expected that [some] months [my particular project] will not work. I never expected to have an interpersonal issue affect my grad career or academic career.

The inappropriate situation was difficult to handle; she was unable to find someone or somewhere to turn to address the dynamic appropriately. She described the responses from her mentor about this and other issues as providing “no real support.” The graduate school had no official HR department to support students. In short, she had no venues through which she could seek redress.

Trainee 4, meanwhile, spent a great deal of her time in the lab managing students. She reported that she was actually running the lab since her mentors in graduate school and during her postdoctoral fellowships were not managing their lab personnel. Her mentors either did not have technicians and lab managers or they had delegated them to other tasks, leaving the personnel and lab management up to her as a senior postdoc. She expressed that her mentor did not positively view her level of productivity; and that she told her: “My papers don’t get written because I’m managing all of your people.” In short, she was dealing with interpersonal issues and mismanagement of people in the lab; she described trainees as being treated like “grunts” and as being “disposable.”

Additionally, Trainee 4 had several experiences in graduate school at another institution, and her postdoctoral fellowship at the research site deterred her desire to pursue academic science. During graduate school, she took a leave of absence for two years to take care of a sick parent. She found it difficult to return to the program after the leave of absence, confronting many obstacles upon her return, including being judged by faculty and her peers for leaving. Similarly, during her postdoctoral fellowship, she said no one provided guidance or pointed out resources to help her deal with personal and family issues, or difficulties arising out of the ebb and flow of success that comes with conducting experiments.

Finally, Trainee 4 perceived that she had been used by two faculty members during her postdoctoral fellowship. She had been paid through training programs and a grant received during her postdoctoral fellowship. When her grant was ending and her mentor would become responsible for paying her salary and lab costs, he told her to find a job and said she should seek employment in another faculty member’s lab because she probably could not run a research lab herself. One week before she was supposed to start the second postdoctoral fellowship, she received a fellowship grant from the American Heart Association, and her mentor stated to her that he wanted her to stay on under his purview. It seemed, she said, that he wanted her to continue having her produce under his lab and name as long as he did not have to pay for her to do so. Trainee 4 experienced a tug-of-war between the two faculty members that lasted for a full year, with both requiring her to do work for them, creating a situation she described as awkward and stressful. She believed mentors were supposed to guide their trainees to reach goals and to establish a strong career trajectory. Her experience, however, was that the mentors were more concerned with their own names, publications, and grants than with providing support to young scholars.

One faculty member, Faculty 3, anticipated and acted to prevent conflicts, particularly with the company she was running outside of the institution. When any student or postdoctoral fellow simultaneously worked for her company, she appointed an ombudsman to serve as a resource for these students and postdoctoral fellows in case they felt exploited or encountered any other issues. She noted that policies existed stating that students could not be exploited, but she claimed the policies had no practical effect–they were more of a formality in place to assure that the institution or the organization would not face a lawsuit. The ombudsman she employed provided mediation, and she expressed that the presence of having someone acting in that role created a much more cohesive and pleasant environment for trainees.

#### Productivity suffers due to a lack of project management

Faculty mentors attributed productivity to successful project management and to efficient and ample dedication of the individual’s time and effort. Trainees and administrators expressed that they felt productivity was hindered by a lack of direction from mentors and often of the project itself. Faculty 3 selected projects for trainees that were appropriate for the amount of time available for training; “We always choose [a project] that’s really high risk, high reward, middle, and then oh yeah, that’s no problem [like one] building on 5 years of data…[The one undertaken is the latter so] there’s never a risk for the trainee.” She told a few stories about labs that did not share her approach. For example, one faculty member was well-established and had funding that afforded supporting 15–20 people. A postdoctoral fellow had been in a position for just three months, but that individual was let go because the faculty member did not think the trainee was producing fast enough. In another lab, two or three postdoctoral fellows were put on the same project to compete against one another, where the “victor” got the “spoils” to assure the faculty member would have a paper published with alacrity. Faculty 3 regarded this type of training as “treating people like commodities” and as providing less of a training environment than a production facility.

Faculty described issues with productivity within the context of thinking trainees needed to spend more time and effort on a project–they needed to persevere and “keep plugging away,” and if they could not “cut that, …they have to go into other areas of science” (Interview with Faculty 1). The impression Trainee 9 said was received from faculty was “either you get out in 5 years or you get out in 8 years, and if you get out in 8 years, the faculty’s looking at you going like, ‘This one probably needs to work at the FDA (Food and Drug Administration) or be a patent lawyer or something.’”

This type of message was discouraging to trainees. Many considered productivity and success in part as the luck of the draw of the project to which they were assigned, and experiments would not necessarily work or contribute to answering and evaluating the research question. This was indeed the case for Trainee 2—the experiments assigned simply did not work, and it was not possible to replicate previous results. This individual had been working with another post-doc who got a job and left, and he had no one to help him maneuver the project’s issues. One would assume that in such a case, the mentor would have stepped in to guide, train, and advise him, providing additional resources during this time of transition, at least during at certain points along the way; however, the trainee was left to struggle through these difficulties alone. He said he felt the mentor was disengaged with their training–the mentor’s focus was on his or her own papers and grants. Eventually, in a performance review from his mentor, he was judged as not making any progress, and the evaluation ended with the message of “do more and do better.” The project was eventually dropped, and a new assignment went well, which meant he could start looking for a job and left shortly thereafter. Still, Trainee 2 was frustrated and said he felt like he had wasted 1.5–2 years of training due to a lack of personnel support and project management.

Trainees expressed that, when they felt they were not being trained or supported by their faculty mentors, they turned to administrators for guidance. The administrators, however, were not poised to provide specific research or grant direction. This was especially apparent when the trainees were required to submit a grant with no direction from their mentor and were “left to drown really,” according to Administrator 1.

Postdoctoral fellows were not given the proper training for the grant seeking and application process. As Administrator 1 explained, “there’s obviously a lack of support by either the PI or just the whole process in general.” The administrator said she saw many grant applicants re-submitting their grants after they had received rejections, and many of the re-submissions were not funded either which led them to feelings of failure. The faculty mentors’ perspective was, in general, “These are post-docs, so they should know how to do this now.” However, Administrator 1 noted that trainees often were not taught grant application skills while in graduate school, and their educational and professional training likely did not include the complex skill of developing grant submissions.

Trainees in the focus group said that workshops on grant writing would have been helpful. Though they found value in having junior faculty speak at workshops about career sectors, getting a job, and topics those faculty members had been encountering, the group wanted to hear from senior, more established, faculty members regarding grant writing (or creating lab budgets, dealing with personnel, etc). Trainee 2 especially wanted to hear “from someone who’s done it and who hopefully has done it successfully …The engagement from [senior faculty] is extremely low.”

#### Faculty attitudes and disengagement unfavorably affected trainees career desires

The negative demeanor and attitude of faculty members had an adverse impact on trainees who described them from behaving unpleasantly in seminars and group meetings, or to colleagues and trainees, to being unable to deal with interpersonal issues, which were not work-related but that at times affected a trainee’s progress. Trainees labeled some faculty members as “unpleasant,” “extremely racist,” “misogynistic,” “dismissive,” “defensive,” “hostile,” and “being a jerk.”

All faculty and trainees mentioned seminars as critical, important, and desired to support training and camaraderie. Faculty members regarded seminars as an important way of training PhD scientists in other research techniques and technical perspectives, and in how to relate to audiences by providing examples of effective presentations and fielding questions.

However, the trainees and administrators noted that faculty members represented a poor example of the scientific engagement the trainees were supposed to be learning. Multiple trainees said faculty often did not attend departmental seminars, even if the speaker was an invited guest. If they did attend, many did not pay attention, but would instead do work or check phone and email messages during the entire talk. Faculty advisors were also described as disrespectful, condescending, or unwelcoming to collaborators and guests; trainees perceived them as hardened and acting out of insecurities. These attitudes contributed to poor communication in the seminar because many senior faculty were not listening to or hearing what others were saying, but they were trying to formulate responses while another was still talking, or they were becoming defensive during discussions.

Multiple trainees and one administrator spoke to the consequences of disengagement when mentors did not connect trainees through networking to colleagues and speakers. Trainee 4 regarded this disengagement as a shortcoming of the faculty mentors and said they typically kept to their own conversations with people at meetings or conferences. She continued by saying the mentors often talked about networking and spider web-like connections, but there was no guidance, assistance, or introductions to aid the trainees in establishing and using these networks.

### Perspectives on career sectors and paths influencing trainees’ perceived expectations

Career development workshops were viewed by trainees as helpful for learning about career sectors and opportunities, as well as non-technical skills needed for establishing and building careers. These workshops were most often requested and planned by trainees but were not funded or usually endorsed by faculty or departments. Over and over, trainees mentioned not having enough workshop sessions to learn about the myriad of existing career sectors and opportunities.

According to trainees and administrators, if there were career path workshops, too much emphasis was placed on faculty appointed positions or skills only needed at research institutions. Administrator 1, for example, summarized the issues highlighted in this area by both administrators and most trainees:

[Faculty are] teaching [trainees] a narrow path, and part of it has to do with how NIH views successful research students and post-docs, because if [trainees are] not doing a faculty-appointed or a position of research at an academic institution, they’re looked upon as not quite making it or that the training grant is not successful as it should be…

**Fig 4:**
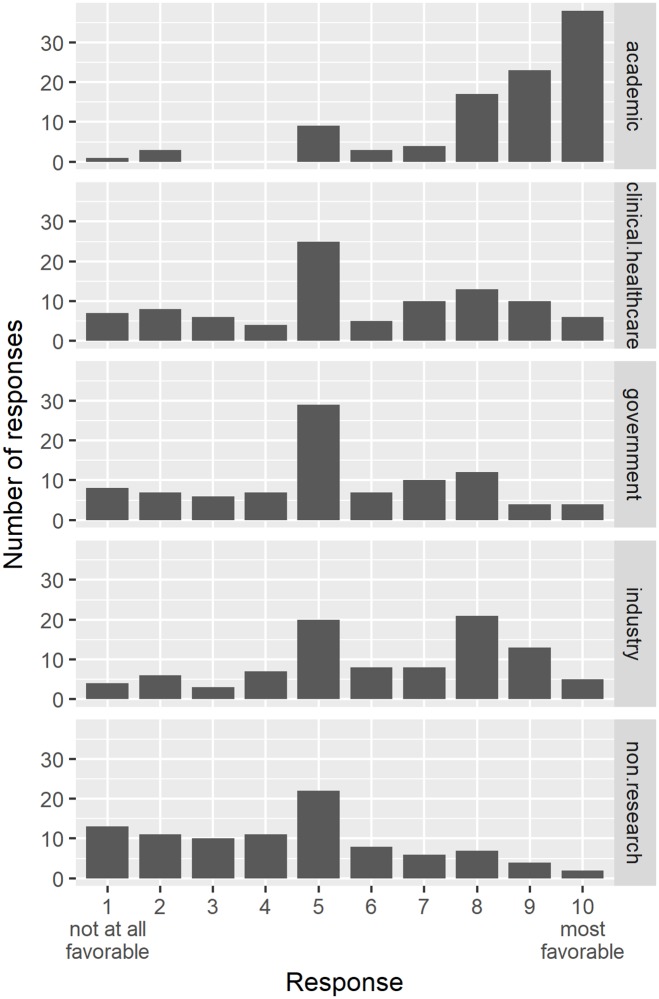
Mentor’s encouragement of career sectors. This figure shows the mentor’s career sector preferences.

All trainees, faculty, and administrators stated in interviews that the intended career path for biomedical science trainees was in academic research, which strongly influenced biomedical PhD scientists and was made obvious in formal settings, such as career development workshops, and informal settings, such as hallway conversations. Fig 4 shows how trainees perceived the levels of support–favorable or unfavorable—from mentors, for each career sector.

When Trainee 4 was in graduate school, her advisor told her: "Go academic. Everybody goes academic. Nobody does anything else.” Two other focus group participants agreed that the expectation was to enter academia and that faculty said and implied it was the way all trainees were “supposed to go.” Administrator 1 emphasized that the faculty pushed academia and did not talk about industry, government, or any other “avenues.” Administrator 2 also perceived academia as the career path faculty expected trainees to follow and that their “perception is, ‘You get this PhD to do this kind of work.’”

Not only did faculty expect trainees would pursue a career in academia, but they said they viewed other sectors as inferior. Faculty 2 said he thought the perception was that faculty felt that if a student went into industry, that person’s abilities were inferior. He did not agree or believe this to be true, however, at least for himself. Faculty 3 also thought perceptions and expectations bent strongly toward academic faculty careers and that an air of elitism was attributed to academia by faculty and the general public. Faculty 1 said she believed such perceptions and expectations regarding academia were old and obsolete, but she also admitted that those who entered non-academic careers were viewed as failures and embarrassments to the profession. While Trainee 10’s advisor did not speak negatively about non-academic career sectors, he expressed a definite bias asserting that academic faculty were of the highest caliber, and the order of the hierarchy of importance was academia, then government, then industry, and then other careers.

Trainees repeated that their mentors encouraged them to pursue an academic track, but a few said that, when they frequently discussed pursuing another direction, the mentor eventually stopped discouraging them and tried to provide direction. Faculty 2 was very open to supporting mentees in careers in all sectors. He stated he was not sure if that was because (a) he was foreign and had not received the same messages as his U.S. colleagues about academia being the preferred option, (b) he had recently come out of training and begun his career and maybe messages to trainees were changing, or (c) he was in a department and research area heavily tied to industry, where it was common for people to move back and forth between the sectors or to collaborate. He said he hoped advisors had become more open-minded than in the past about career options, because “really nothing is more important than having a successful trainee in the end. A successful and happy trainee. That’s the bottom line.” He explicitly acknowledged, however, that often, if a trainee wanted to go into another career sector, it could become problematic for an advisor outside of that sector whose experience was limited to academics to know how to train or guide them toward this goal. He stated that, when he learned a trainee might want to go into industry after a postdoctoral fellowship, he would send the trainee to a biotechnology industry organization conference.

Anxiously contemplating her career path, Trainee 3 said receiving more open support and guidance from her mentor would have helped her feel better about the entire process. In this same vein, Administrator 1 said she had heard from many trainees, especially females, that academic positions were pushed as the only valid professional paths. She noted that mentors rarely talked about industry, government, or any other venue for professional development; students were openly discouraged from pursuing allegedly “non-research” careers like “technical writing, patent law.” The only people who went into industry from Trainee 9’s recollection were those who had mentors with a start-up in town, so the trainee would transition from training in the lab to working for the mentor in another setting. Otherwise, he said, nobody he knew had landed a job in industry. Trainee 7 summed up the thoughts expressed by many trainees when she stated:

They’re teaching them a narrow path, and part of it has to do with how NIH views successful research students and post-docs, because if they’re not doing a faculty-appointed or a position of research at an academic institution, they’re looked upon as not quite making it or that the training grant is not successful as it should be.

Administrator 2 said she found that this tension regarding academia and the NIH might be mitigated if a path were created for trainees “who want a higher degree, but don’t want a PhD.” There was no admittance into a master’s program, but if a graduate student left the PhD program midway and had made enough progress, he or she could leave the program with a master’s degree. Thus, having a master’s from a PhD institution was seen as a “fail-out.” She said she thought many graduate students in her program would do a master’s level program, particularly because employment options would be greater with that level of training than with a bachelor’s degree, and such opportunities would be a better fit perhaps for those individuals not desiring to follow the PhD path. In the programs at the research site, graduate students had the option of earning their master’s degree as they advanced to candidacy or on the way to a PhD, but mostly the master’s was designated only for those “who would not be continuing on to the PhD,” adding to the stigma of failure.

## Discussion

The research study considered personal, environmental, and experiential factors, as guided by SCCT, which influenced trainees’ career paths. Many factors influence PhD scientists’ career sector preference and job search process, but the most influential were relationships with faculty, particularly the mentor advisor.

Nearly all trainees, faculty, and administrators focused on the great impact mentors and advisors have on trainees’ career path choice, and on how trainees felt about this choice or where they ended up. Overall, interactions between trainees and their mentors and other faculty were very positive. The mentors with the highest regard cared about trainees personally, supported their career path goals, challenged them intellectually, and had compatible personalities.

Expectations influenced trainees’ preference for certain career sectors. Faculty did not feel their bias toward academia was overt or visible, but trainees and administrators felt the message received from faculty had this bias. Faculty viewed other career sectors as inferior.

In general, faculty often do not talk about, or at least not favorably, other sectors, which contributes to the trainees’ lack of awareness [24, 38]. With the participants in this study, the blind spot or narrow focus was partially due to people discussing the path they took and knew [1, 50]. Faculty are under pressure, especially from grant funding sources (of which the largest is the NIH), to maintain the definition of success for a biomedical PhD scientist as entering a full-time tenure-track academic faculty position at an R1 institution, receiving continuous and high grant funding, and publishing often in high-impact journals.

Faculty continued to use phrases with negative connotations such as “alternative” careers [6], and paths. Past trainees in academic careers were more positive about interactions with their mentors, whereas those who entered non-academic areas were less positive. Regardless of the pro-academic bias, some trainees expressed interest—as they should according to Alberts et al. [5]—in non-academic careers, and faculty helped them apply and network, or were generally supportive and encouraging of the chosen career path. As the mentor is such an integral part of the decision-making process, this is even more reason for broadening the definition of success, being more open to other career sectors, and altering how they talk about these sectors.

Relationships with mentors were less strained when faculty appropriately handled personnel issues within the lab, and when the lab was adequately staffed with employees to manage ordering, equipment schedules, or technical work, allowing graduate students and postdoctoral fellows to focus on their projects. When these tasks were left to senior students or postdoctoral fellows, it took time away from their projects, lessened productivity, and created resentment and negative feelings. Permanent career scientists were lauded by Benderly et al. [51] and Teitelbaum [40] as giving indispensable support to the research lab, and by providing these employees with reasonable salaries and benefits, the competition for some academic positions would be reduced [9].

Some labs were too competitive, and if mentors interviewed encouraged a “dog eat dog world” mentality instead of teamwork and collaboration, trainees were negatively impacted. Trainees felt success was often due to the project chosen or assigned, while faculty thought that trainees simply needed to work longer hours, work harder, or think more strategically. Perseverance was identified by faculty as a required characteristic for biomedical scientists wanting to enter academia; however, trainees’ description of their experience illustrated perseverance regardless of ultimate career.

Those trainees who approached faculty for guidance and did not receive it turned to administrators who were unable to appropriately direct them. Faculty engagement with committee members and departmental or program faculty was beneficial and impactful. Committees were recommended by Fuhrmann et al. [1] for overseeing trainee career development. Collaborations with other faculty members were a highlight of some trainees’ experience. Trainees underutilized faculty as resources and for support. These experiences and the thrust toward academic careers impacted whether trainees saw academia as a favorable career option worth pursuing.

During seminars and other planned activities, faculty were poor examples of scientific engagement, as they often conducted other work, answered messages on their laptops or mobile devices, or asked presenters difficult or impossible questions during seminars. Often they did not even attend planned activities unless absolutely required or unless the presentation discussed a directly related area that would benefit the faculty member. Career development workshops by established scientists with considerable experience or success in the field were desired, but trainees found it difficult to get senior academic faculty to commit to presenting even one session.

Internships and shadowing experiences were found by trainees and faculty to be beneficial for gaining a larger perspective on how the science fit together, or for determining if industry or other career sectors were ideal for a trainee. However, faculty could not pay the trainees’ travel costs or stipends for internships because of grant funding constraints and the lack of any other available funding sources. Trainees were also under faculty and departmental pressure to be in the lab as much as possible, and these experiences would extend the amount of time needed to complete degree requirements or training.

Intended careers varied, and those who always intended to take a non-academic path were not viewed favorably. Many changed their minds as they progressed in training and learned more about what academia entailed and what other options were available.

Success in obtaining the intended career was attributed to both internal and external factors, but failure was attributed to external factors undermining the individuals’ efforts. Motivation was met by conflicting outside forces, reducing the number of available career sectors. Perceived and real barriers contributed to outcome expectations, reduced the number of choices individuals had, and impacted the career paths trainees were able to navigate. SCCT shows personal, background/environmental, and experiential conditions and events as regulating individuals’ goals, actions, and performance domains. Though the SCCT model emphasizes that trainees are not passive in their career paths, this study revealed that environmental or “background contextual affordances” can mitigate or erode trainees’ interest, efficacy, and obtainable domains. Background contextual affordances and contextual influences by others can adversely or beneficially affect trainees. Several researchers applauded a change in career preference as beneficial to the trainee and the system as a whole, and as demonstrating that the training process was working as it was supposed to [24, 28]. SCCT demonstrates the strength of a model considering multiple potentially countercyclical aspects impacting one’s training experience, career expectations, and career paths.

### Implications for practice

Networking was crucial to career searches. Trainees need to learn how to network, but faculty must also be willing to boost their efforts. During conferences or society meetings, faculty should take an interactive and intentional approach to introduce their trainees to colleagues or encourage colleagues to attend their trainees’ presentations. During departments and centers’ seminar series, faculty rotate inviting guests to visit and provide a seminar, and during the visit the inviting faculty member acts as a host. By allowing a trainee to help plan the visit, provide transportation from the airport, join the guest for meals, or just meet with the guest, the faculty member would be providing key networking opportunities. Even if the mentor is not hosting an outside speaker, faculty often meet with guests from relevant scientific areas and could include trainees.

The definition of success needs to be redefined to include non-academic career paths, particularly as career prospects often follow a winding pathway rather than a linear approach [7]. While public funding should support the training of biomedical scientists for academic research, the NIH should acknowledge and promote scientists in non-academic sectors as well. High caliber biomedical scientists are crucial in government, clinical healthcare, industry, and other career sectors too, and PhD scientists should be able to pursue careers in those sectors without stigma. Expanding the accepted sectors and changing the culture would be ideal. Additionally, grant review committees should consider including non-academic scientists to proactively strengthen perspectives and confront biases.

Academic and career goals need to be clarified early in the training process with the faculty mentor. Based on the findings, it is clear that interests and goals develop and change throughout training, and this must be communicated without apprehension to the mentor to allow the mentor the opportunity to minimize barriers and facilitate growth and success. Faculty can train and support graduate students and postdoctoral fellows in directions appropriate for their aptitude and interests, instead of creating an additional hurdle in their path. Additionally, graduate students and postdoctoral fellows should continue to ask questions of their mentor, faculty committee, lab mates, and other colleagues to determine if academia or another sector is right for them [52].

### Limitations of findings

The main limitation of the findings is their generalizability and transferability. Participants were recruited by sampling a specific program at one highly selective institution. Their experiences as written in the findings may not be indicative of all biomedical PhD scientists at this research site or at other institutions. However, the literature indicates that similar issues are found at institutions throughout the United States and around the world, and these overlapping aspects may inform and benefit other populations.

Contact information was readily available for most past trainees who were in academia, as those institutions’ web pages are available and prominent. Past trainees in non-academic careers were more difficult to find, as large companies, healthcare facilities, independent consultants, and other employers may not make contact information publicly available. Data collection was conducted over a summer, when many people in all career sectors are on vacation, otherwise disengaged from work, in the middle of grant submissions, sabbaticals, writing books and publications, having elective surgeries, or otherwise unavailable.

This research study was also limited because it relied on self-selection for the interview phase and on volunteers contributing documents for review. Several prospective faculty and administrator participants did not want to participate for fear their feedback would highlight a negative training environment or that they may be identified. This may have been true of current or past trainees as well. As non-academic sectors are still regarded as inferior, perhaps this impacted whether scientists would be willing to participate in a study on navigating different career paths.

Additionally, regarding the theoretical framework informing the study, the factors incorporated in SCCT encompass more roles and intersections than can be discussed and understood here. Though the researcher has taken lengths to inquire about the many factors that SCCT accounts for, using them to inform questions, coding and analysis, there may be factors, which have been overlooked or are not yet discovered. SCCT considers individual characteristics and experiences, interactions with others, and external influences, which, if taken comprehensively in their entirety, represent a scope broader than any one research study can discover and explain. Fig 1 presents these factors and their intersections to the extent they were identified and incorporated into this study.

### Areas for future research

There has been an insufficient amount of research regarding non-academic careers, the training and support biomedical PhD scientists receive, and the experience and process of navigating a career path. Fuhrmann et al. [1] recommended a shift in the academic culture to embrace the branching science career pipeline, of which little is known. Future studies could investigate whether advisors mainly discuss academic paths due to their personal experience, knowledge, and networks.

This same case study could be replicated at other programs or institutions or use different qualitative approaches. Some factors identified by SCCT should be investigated in more detail at a larger scale. In particular, it would be beneficial to research how individual inputs impact career path choice and navigation. If this specific program for promising graduate students and postdoctoral fellows at a large selective institution could improve its career development opportunities, much more could be learned from other programs or institutions with fewer resources and connections. Perhaps others are using novel career development training and strategies.

More research conducted internationally is warranted to discover what training and support is provided to biomedical PhD scientists in other countries. Additionally, as many other countries have different definitions of success and failure, different needs and availability for academic positions, and may train students differently, expanded research should be undertaken to find where issues intersect and to discover solutions that could be applied in the United States.

## Conclusion

Unlike prior studies, this work used an open definition of success based on the SCCT model, incorporating trainees’ intentions, interests, and goals; their reported training and career navigation experience; and their perception of whether they were prepared for a career and satisfied in the sector or position obtained. Capable and successful biomedical PhD scientists are engaged in varied professions in both the academic and non-academic sectors. Kemp et al. [30] and Lee et al. [15] agreed that scientists who have the technical skills, discipline, intellectual rigor, passion, and curiosity to make a significant contribution to biomedical science should be encouraged and trained to do so. The skills and training needed for careers outside of academia have been rarely researched, and this study contributed to the knowledge of essential career development for the large proportion of graduate students and postdoctoral fellows from science, particularly biomedical science, who enter non-academic careers.

Recommendations for reconstructed career development opportunities and information emerged from the data, chiefly from trainees and administrators. This is appropriate and expected, as trainees and administrators were most interested in non-academic career options, while faculty were most interested in academic ones for themselves and their mentees. The findings also promote a career pathway where one can practice and conduct biomedical science in many professions and can contribute to the reconstruction of a career structure to provide young biomedical scientists with the hope and preparation necessary to become leaders in science-grounded careers. Universities must encourage and facilitate creating these skilled scientists who aspire to a variety of career opportunities [30, 53].

## Supporting information

S1 Appendix Research design. The supplementary appendix details the full research design and includes all templates used in the study.
(DOCX)
