# Supplementary material for: Navigating the path to a biomedical science career
Source: PLoS One. 2018 Sep 7;13(9):e0203783. doi: 10.1371/journal.pone.0203783 (PMC6128638; doi:10.1371/journal.pone.0203783)
Supplement: S1 Appendix — The supplementary appendix details the full research design and includes all templates used in the study. (DOCX) [file pone.0203783.s001.docx]

# S1 Appendix

# Research Design

This study was conducted to gain a better understanding of the career search process of biomedical scientists. The purpose of this case study was to understand the process through which biomedical PhD scientists at a large southeastern tier 1 research institution are trained and supported for navigating a future career path. In addition, the study sought to determine whether career development support efforts and opportunities should be redesigned to account for the proportion of PhD scientists following non-academic career pathways. Though all career paths were reviewed in literature, non-academic careers were the focus of this research study.

This supplement of the larger study describes the research design and approach that aligned the researcher’s methodological choices with the research questions and selected theoretical framework. First, the research approach and tradition are explained. The last section details the research process, including how this study was conducted, ethical considerations, trustworthiness, and possible limitations.

## Research Questions

The central research question driving this study is: How does a southeastern tier 1 research university train and support its biomedical PhD scientists for navigating their career paths? Data collected illuminated how the process influenced the outcome of the job search and explored other factors and experiences contributing to career paths sought and chosen. A practical sub-goal of this study was to uncover suggestions and ideas about skills that should be cultivated to benefit and prepare biomedical PhD scientists for employment after graduation.

## Research Approach

As explained in the literature review, biomedical PhD graduates and postdoctoral fellows are pressed to enter academic research by their faculty mentors, institutions, and funding sources, but there are not enough positions to sustain the number of graduates. The NIH, a federal funding agency, is the main funding mechanism of biomedical science graduate students and academic research with the goal of ensuring there is an adequate biomedical workforce to sustain the future of scientific research [28]. Most studies have reported on trends in the outcomes of biomedical PhD scientists through quantitative methods [16, 18-25]. These studies have used the traditional academic pathway as the definition of success. However, the focus of this study is on non-academic career pathways and understanding the process and experience for biomedical PhD scientists, not simply how many employees work in each sector. There has been little qualitative research conducted on the job search experience of biomedical PhD scientists, especially on the skills and training that would have best aided them in preparing for a career outside of academia.

## Research Paradigm

Interpretivism conceptualizes the world as having more than one reality and not as an object that can be discovered, measured, and determined; rather, the world is constructed by human beings perceptions and interactions [54-55]. While positivists and post-positivists see the world as objective and static, interpretivists see the world as subjective and dynamic. Instead of inquiring about facts and certainty, interpretivists seek answers to *how* and *why*, which is more suitable for qualitative research than quantitative. Interpretivists, particularly social constructivists, emphasize human experience and meaning making. Therefore, social order and historical context are important aspects of interpretivism in the process of making sense and meaning; the paradigm asks and answers different questions than positivists and post-positivists. Interpretivism asserts that there is value in the co-construction of insight and meaning through dialogue and reflection between researchers and participants, and this paradigm provides richness and depth to the data. For these reasons, this research study uses the interpretivist-constructivist paradigm, which naturally aligns with a qualitative research design.

## Research Design

A number of differences exist between qualitative and quantitative research, particularly the type of question(s) being asked. Though research problems addressed by either qualitative or quantitative studies may be similar, the specific research question(s) should determine which type of study a researcher should undertake. Quantitative studies generalize the aggregate behavior of populations by asking *how many*, and qualitative studies see issues as complex while asking *how*, *what*, or *why*. This study focuses on *what* and *how* questions in efforts to explore the career experiences of biomedical scientists through the interpretivist-constructivist paradigm; it does not seek to explain or generalize results to an entire population. In qualitative studies, a researcher may only have a rough idea about a phenomenon when beginning his or her study; in short, this research approach does not require, or often use, a hypothesis to begin or direct the study. Qualitative research is also more exploratory in nature and may begin with vague inquiries that are refined or that change over the course of the study as the researcher discovers, explores, interprets, and constructs understanding, meaning, patterns, themes, and theories. This approach is inductive and asks the researcher to remain open throughout the research process to allow the data to shape the study as meaning is interpreted and constructed.

The role of the researcher using social constructivist perspective is to minimize bias and acknowledge positionality while seeking meaning, in this case, meaning that biomedical scientists have made of their career path process. The goal of this study was to explore multiple realities and perspectives through interactive dialogue, analysis, and reflection. The researcher acted as a facilitator of the interpretation to allow for the emergence of themes, patterns, and meanings. However, the researcher strived to remain neutral to interpret data responsibly and without imposing bias on the participants or data. Handling all interviewees with respect and discretion was essential to minimizing bias; the researcher took all precautions to protect participants when sensitive information was revealed. To minimize risks to individuals and their organizations, names and identifying information have been hidden in the findings and analysis. The details explaining how the researcher protected participants and minimized risk is discussed at the end of this chapter, following the descriptions of the research method selection and data collection processes.

Qualitative research uses a larger variety of methodologies than quantitative studies. Qualitative methodologies include case studies, conducting interviews or open-ended questionnaires to create a narrative, phenomenology, observation, content and context analysis, first-hand experience, and collecting field notes and reflections. This study employed the case study method; the rationale and reasoning for choosing this method is detailed in the next section.

## Research Tradition: Case Study Method

The case study is a research strategy exploring a phenomenon within a context by using multiple sources of evidence [45]. The case study approach allows for a detailed and in-depth look into data over time through multiple sources of information and data collection. Each data source type includes its own sampling, data collection, and analysis strategies [46]. A quick overview of case study methodology is provided below, followed by a more detailed description of the different approaches to case studies and a discussion of the reasons for choosing a particular case method for this research study.

The case study method or approach has been used by many disciplines and researchers for decades; however, the use of the qualitative case study approach has been used increasingly in the past 15-20 years and is gaining reputability. Many researchers could be included in a discussion of the rise in the case study as methodology, but two researchers in particular are instrumental to its acceptance and promotion: Yin and Stake.

Among the different scholarly perspectives of the case study approach, Yin broke methodological ground; he was foundational in outlining and describing the basic set of research designs for conducting single or multiple case studies. His design is the most frequently used approach. While Yin’s work has been employed with greater frequency, Stake adopts a different philosophical approach that is equally important in the field of case study research. Yin [45] highlighted the importance of qualitative work even though the result of the methodology is not generalization. Stake and Savolainen [56] also reiterated that generalizations are not the goal of case studies; rather, case studies aspire to know one particular case as it is, with differences and uniqueness emphasized. Stake and Savolainen [56] thoroughly delineated the justification for using the case study strategy to conduct research, describing the approach as a “naturalistic, holistic, ethnographic, phenomenological, and biographic research method” (pp. xi).

The case presented in this study includes a broad range of factors, namely those identified in the SCCT theoretical framework outlined in the first chapter. Yin [57] defined case studies as explanatory, exploratory, or descriptive, while Stake and Savolainen [56] delineated case study variations as instrumental, collective, or intrinsic. This study is an exploratory case study, seeking to understand the process through which biomedical PhD scientists navigate non-academic career paths.

The case study method allows the researcher to investigate the case in depth, while cross-analysis of individuals’ experiences supports the emergence of themes and patterns through inductive reasoning to explore a contemporary phenomenon. As the goal of research is to learn more about specific subjects, people, organizations, or issues, the researcher must rely on and use the method(s) that will make understanding and portraying the data most effective. The case study approach is not best for all studies, but it can provide a depth of understanding that cannot be as easily discovered by some other methods. For the research goals and questions this study addressed, a case study analysis provided more depth of understanding the search for a career and positions of biomedical PhD scientists than, for example, a narrative approach.

## Participants

This case study provided an in-depth analysis of the individual experiences of biomedical PhD scientists from the research site, together with an analysis that identified themes; it contextualized the experiences of the scientists to aid in the interpretation of the meaning of the case. The unit of analysis employed was a “case” consisting of an individual, an organization, an event, another entity less well-defined than individuals, or multiple individuals as a multi-case [45]. The choice to use the case study method related to the way the initial research questions and research purpose were defined. To understand the job search process and experiences of biomedical scientists, the researcher conducted a single case study at one research site with multiple individuals comprising the units of analysis. Typically the sample size for qualitative studies is fairly small, and the researcher may be familiar with the characteristics of the participants. This case study focused on biomedical PhD scientists in a specific training program supported by the NIH. Though this study’s intent was to learn about non-academic career paths, data were also collected about and from academic researchers for contextualization and cross-analysis.

## Research Site

The specific research site and sample characteristics were selected for multiple reasons. First, the focus of NIH training grants is to train biomedical scientists to go into academic research; however, many do not intend to do so or do not enter academic positions. The training program examined at the research site is one of the largest and longest running in the United States, with trainees spanning many degree areas and most departments. This program also supports and trains both graduate students and postdoctoral fellows, which is not typical of many NIH training grants. At the time this study was conducted, the program was supporting eight graduate students and seven postdoctoral fellows for a total of fifteen trainees per year. Though this number has varied over the years, this is the average number of trainees supported per year. Some trainees are also supported for multiple years. The training program has existed for more than 40 years, so its longevity, the combination of pre- and postdoctoral trainees, and the number of trainees who have been supported by the program together create a large sampling frame.

## Sample Characteristics and Procedure

Since several types of data were collected and the scope of the case study contains both depth and breadth, multiple sampling strategies and characteristics were included. In addition to initial and broad convenience sampling, quota sampling was employed in later stages of the study. The following four groups of participants comprised the sample:

### All trainees.

All past and current graduate students and postdoctoral fellows who have been supported on this NIH training grant, and have current contact information available, were contacted. This group included those in training, those who had recently completed training and begun careers, and those who had made several job changes throughout their careers. This group consisted of approximately 300 individuals and is referred to as *All Trainees*. As the data collection with this group was exploratory and helped to create a base sample for the third group, described below, a response rate of 15% was considered acceptable. Most responses came from those currently in training at the institution (*In Training*) or those who had completed training (*Trained*), though the researcher aimed for a representative sample with a mixture of those trained at the institution as graduate students or postdoctoral fellows, males and females, multiple departments and programs, and those in various training or career stages.

Of the 306 trainees who had been part of the training program before or during the time of data collection, 88 either could not be found or did not have current email information, at least two former trainees were deceased, and one was stationed in a remote international location without access to the Internet. There were 214 potential survey participants, and 98 completed the survey. Though all potential survey participants may not have received the invitation, the survey response rate was at least 45.79%, which far exceeds the proposed acceptable rate of 15%.

The age range of respondents was 24 to 67 years of age, with a mean of 47 and a median of 36. Fifty-six males and 41 females completed the survey. Fifty-seven survey participants trained at the research site as pre-doctoral students, 30 trained as postdoctoral fellows, and 11 completed both pre-doctoral and postdoctoral training at the university. These numbers are fairly representative of the biomedical science training program. Just over half (54 respondents or 56.3%) of survey participants reported the academic sector as their current career sector (see Figure 3), but the survey did not take into account those currently in graduate training or completing postdoctoral fellowships at research site or elsewhere, who also marked academic as their current sector. The distribution of prior career sectors (see Fig 2) is similar to the current sectors; however, the total number of responses is greater than the number of respondents, as respondents were allowed to choose multiple prior career sectors.

The final question asked whether the individual would be willing to be included in one of the next phases of the study -- a focus group or interview. One of the main purposes of the survey was to get a sample pool for in-depth interviews and focus groups. More than one-third (37) of the survey respondents volunteered for the next phase of the study. However, only 34 were considered, as three did not leave name or contact information in the anonymous survey. Details of these participants follow in the next two sections on *In Training* and *Trained* descriptions.

### In training*.*

This group included graduate students and postdoctoral fellows who were being or had been supported by the NIH training grant and who were still at the institution. Approximately 45 individuals were available from this group, which is referred to as *In Training*. Six survey participants, who were still in training, volunteered to participate in the focus group. Two trainees were unable to participate due to travel and clinic schedules. Demographics of the focus group participants are shown in Table 1. As the focus group participants are currently in training and are fewer in number, their years in training were excluded from the table. Also, as focus group participants have not yet entered the workforce, the current and prior career sector questions were not applicable.

For document review, *In Training* participants submitted lecture notes from five career development seminars, a research statement and career objectives for application to graduate school, and a personal statement for application to the training program.

### Trained.

Individuals who were supported through this NIH training grant and had completed a PhD program or a postdoctoral fellowship made up the major focus group of the study as *Trained* biomedical PhD scientists.

The researcher aimed to select at least two former trainees from each non-academic sector to interview. There were 13 academic sector volunteers, six from industry, three from government, and two from other or non-research fields. Thirteen volunteers trained at the research site as pre-doctoral students, nine as postdoctoral fellows, and two completed both pre-doctoral and postdoctoral training at the university. These numbers are fairly representative of the training program makeup, so the researcher did not oversample any particular group. Several volunteers had experience in multiple sectors and were selected, even if they were currently working in the academic sector, because of their non-typical career path. Nine former trainees were contacted to schedule an interview, and one person was non-responsive. The demographics of participants in the individual interviews are shown in Table 1.

Table 1*:
Demographics of Trainee Participants in Focus Group or Individual Interview*

| **Trainee** | **Years in Training** | **Pre or Post-doc** | **Sex** | **Current Career Sector** | **Prior Career Sectors** | **Career Sector Interest Prior to Graduate School** |
| --- | --- | --- | --- | --- | --- | --- |
| 1 | Current | Pre | F | Not applicable | Not applicable | Academia, Healthcare |
| 2 | Current | Pre | M | Not applicable | Not applicable | Industry |
| 3 | Current | Pre | F | Not applicable | Not applicable | Healthcare, Government |
| 4 | Current | Post | F | Not applicable | Not applicable | Academia |
| 5 | 1991-1995 | Post | M | Academia | Industry | Academia, Industry |
| 6 | 1993-1998 | Pre | M | Non-research | Industry | Academia, Healthcare |
| 7 | 1990-1998 | Pre | F | Government | Academia | Academia |
| 8 | 1998-2003 | Pre | F | Non-research | Academia, Industry | Industry |
| 9 | 1998-2003 | Pre | M | Academia (non-traditional) | Academia, Government, Industry | Healthcare, Industry |
| 10 | 1981-1988 | Pre | M | Industry | Academia, Industry | Academia |
| 11 | 1994-1998 | Pre | F | Government | Government, Industry | Industry |
| 12 | 1979-1985 | Pre | M | Industry | Academia, Industry | Academia |

### Faculty.

Faculty mentors who had trained graduate students and postdoctoral fellows supported by the main NIH training grant or other grants or means constituted the next group. When this study was conducted, there were 46 faculty mentors across 13 departments or divisions with a wide range of experience. A sample size of 4-6 faculty mentors was considered sufficient for exploring their perspectives on training for career path preparation. Four faculty members volunteered to participate in an interview (see Table 2). For the document review, one faculty member submitted a personal statement for a grant and a career development presentation.

Table 2:

Demographics of *Faculty* Participants

| **Faculty** | **Rank** | **Sex** |
| --- | --- | --- |
| 1 | Professor | F |
| 2 | Assistant Professor | M |
| 3 | Associate Professor | F |
| 4 | Associate Professor | M |

### Administrators.

Administrators of the departments, other NIH training grants, the Office of Postdoctoral Affairs, and the Graduate Programs Office were included in the *Faculty and Administrators* group to learn about the training process and opportunities available for graduate students and postdoctoral fellows outside of the research laboratory.

Thirty associated program administrators at the program management and program director levels were contacted. Several of the associated program directors overlapped with the program faculty mentors but none volunteered to participate; thus, there was no issue, which set of interview questions to ask them. Two program administrators elected to participate in an interview (see Table 3). One administrator submitted one new career development course description.

Table 3:

Demographics of *Administrators* Participants

| **Administrator** | **Time in Position** | **Sex** |
| --- | --- | --- |
| 1 | 2 years | F |
| 2 | More than 10 years | F |

## Recruitment and Access

This study was discussed with the director of the training program and verbal permission was initially obtained to use the database contacts. The director of the training program was serving as the gatekeeper of the database contact information and his approval was necessary to gain access to database. Written permission from the gatekeeper (Appendix A-all appendices mentioned within this supplement appear at the end of this document) was obtained prior to the submission of Institutional Review Board (IRB) application. Once the database of contact information was provided to the researcher, it was contained on a server under two layers of password protection with access available only to the researcher.

The researcher was constantly cognizant that, because she formulated questions, interpreted data, and reported findings, an informal position of power may be present. Handling all participants with respect and discretion was key to minimizing bias and also to protecting participants when sensitive information was revealed. Access and permissions; a brief written description of the intended case work and an extensive plan, when requested, with plans for report distribution or an opportunity to review and edit; and ways to provide anonymity and make changes were crucial as the researcher explored each case in depth and made sure that risks to privacy were curtailed [56].

To minimize risks to individuals and their organizations, names and identifying information have been hidden from the findings. In order to minimize psychological risk, participation was voluntary and participants were informed of their right to withdraw from the study at any time. No incentives were offered. Participants received an informed consent form (Appendix B), which explained the purpose of the research study, its significance, how data was to be collected, managed, and stored, and the procedures for protecting the participants. Transcripts of verbal data collection were sent to participants for review. The participants had an opportunity to clarify or remove any statements they did not wish to have included. Participants also had the opportunity to withdraw from the study at any time and to have any data collected immediately destroyed. The researcher received approval for the study from the IRB at Northeastern University before contacting any participants. The approved IRB was submitted to the research site for permission to conduct the study, which was received.

An email was sent to the *All Trainees* group describing the purpose and scope of the study, as well as potential risks and benefits, to recruit participants for the initial phase of the study (Appendix C). The unsigned consent email detailed consent, privacy and confidentiality, and the option to withdraw from the survey. The end of the consent email included a link to the survey. A thank you message was sent to participants upon survey completion (Appendix D). A reminder email with a link to the survey followed within a week (Appendix E) with a forward of the previous invitation and consent letter included. The final question of the survey asked whether the participant would be willing to engage in the next level of the study.

Those individuals who self-selected into the second level of the study were separated into two groups, those who were currently in training and those who had completed training. Participants belonging to the *In Training* group were subsequently recruited by email for focus group interviews to understand the support and training systems they were receiving, their experience in training, and their perceptions about career paths (Appendix F). The goal was to identify at least six participants through quota sampling; specifically, two individuals from each of three sectors: industry, government, and non-research fields (e.g. consulting or scientific writing). Individuals were selected based on the total of responses to further participation to gain maximum variety in the sample. The *Trained* participants received a recruitment email and consent letter to schedule interviews (Appendix G).

This group was more extensively interviewed in order to collect rich data from scientists who had entered different career sectors, which broadened the scope and provided a more thorough analysis of the issue. Six individuals in a case study is sufficient to gain variety and allow for in-depth interviews with each interviewee. The researcher reviewed individuals from the *All Trainees* survey who self-selected into the sample for the group 3 interviews and screened for the sectors of employment and time since training completion.

Similarly to the recruitment process for trainees, an email was sent to the *Faculty* mentors of the training program describing the purpose and scope of the study, as well as potential risks and benefits (Appendix H). The letter detailed consent, privacy and confidentiality, and the option to withdraw. The emailed letter asked whether the participant would be willing to engage in the study. The researcher selected faculty mentors to interview to gain maximum variety in lengths of mentorship, research and program areas, rank, and gender. *Administrators* of programs, departments, and career development were sent emails to request participation in interviews about training opportunities (Appendix I). This group consisted of a smaller potential sample, and because they were participating for largely informational purposes, the researcher interviewed any and all who agreed to participate. Because this phase of the study did not depend on the other phases of the study, this phase ran concurrently with other phases. This technique was indeed beneficial because, as anticipated, data collected from faculty and administrators helped the researcher shape questions included in the focus group(s) or interviews, and participants were asked to reflect on and respond to data the researcher had collected from the informational, exploratory interviews with faculty and administrators.

## Data Collection

The case study approach is time consuming; it requires the review of a large number of documents and can include quantitative data. Indeed, Stake and Savolainen [56] agreed that researchers using the case study method often collect more data than they actually code and analyze, but the search for meaning relies on identifying patterns and maintaining consistency. The decision to use a case study as methodology inevitably influences the research design, data collection, and analysis. Initial data collection can change the case study plans and protocol and researchers should remain flexible to allow for these changes [45-56]. This section details the data collection process used to conduct the study phases previously outlined.

### Phase 1: survey of *all trainees*.

The initial phase of the study was comprised of a comprehensive online survey sent to all trainees with current contact information in the training program database. The survey resulted in a broad overview of career pathways of 40+ years of PhD scientists who had been supported during a portion of their training at the institution.

The survey instrument is included in Appendix J. It included 19 closed- and open-ended questions spanning multiple factors as identified by the selected theoretical framework and the literature review discussed. The survey did not address the full scope of the sets of factors identified through SCCT; some factors were considered better suited for more in-depth questioning (see Table 4).

Table 4*:
SCCT Factors Addressed in Survey*

| **Sets of Factors Identified by the SCCT** | **Addressed in Survey** |
| --- | --- |
| Individual’s characteristics, experience, background, and interests upon entering graduate school | - Demographics - Interests or intentions prior to graduate school, including desire for an academic or non-academic career |
| Learning and performance in graduate school; skills acquired; the career search; interaction with mentors, faculty, and other students and researchers | - Likert scale responses on faculty interactions - Likert scale responses on career development opportunities during training - Likert scale responses on how training prepared participant for their career |
| Career path and necessary skills and abilities that should have been included in graduate school and postdoctoral training to aid in career development and the transition to work in or out of academia | - Current position - Previous positions - Open-ended listing of career development skills needed |

Google Forms was used to build and conduct the online survey because it was user-friendly, easily accessed by potential participants, tied to the researcher’s Northeastern University email account, and offered an anonymous option. The anonymous option allowed participants to respond to the survey without inserting personal information so only those volunteering to participate in the next phase of the study needed to share personal information. Data can be reviewed within the site as responses to questions or through various data visualization tools, and can be exported in multiple formats for further analysis. The survey was slated to take no more than 12-15 minutes to complete and was piloted with a biomedical graduate student in a different program than those included in the case study.

### Phase 2: focus group with *in training* and document review.

The next phase of the study utilized one focus group. Yin’s highly structured preference for use of a case study strategy recommends conducting a pilot test, which is not a pretest but rather is used to assist in the investigation to develop relevant lines of questions and to provide insight into the issues being studied [45]. The focus group questions were piloted by a former biomedical science graduate student and postdoctoral fellow who was trained in another program. The focus group technique allows for triangulation of data and member checking, which is advised by Maxwell [58] as a way to see different types of responses to compare with others data collection methods to strengthen validity, reliability, and confirmability of findings.

A topical focus group allowed the researcher to bring together people who had experienced similar training to allow the nuances of backgrounds, perceptions, and experiences to come through, which indeed differed from individual interviews [49]. The moderator guided the conversation but also provided more active listening than questioning to allow participants to interact and spark off of one another to co-construct themes together and to validate the analytic process, but not necessarily the data [59].

The focus group allowed the researcher to interview those scientists who were actively in training. These individuals were at the institution and were easily accessed. Members of this group also had not experienced the outcome of the job search nor did they have information on which skills and opportunities would have been useful while in training, as their scope was limited. However, they were closer to their experiences prior to graduate education and generally had more accurate recollections of them. Three to four focus groups are usually recommended until there is saturation or until no novel information is arising; the groups can be used to lay the groundwork for more in-depth data collection [60]. However, with a small group of current trainees and fewer self-selecting into the next phase, one focus group of current trainees was sufficient. In this study, the more in-depth data collection was facilitated through document review and one-on-one interviews.

The focus group session lasted approximately one hour; it proved easier to get graduate students and postdoctoral fellows working in the lab together at lunch time or after 4 PM, so refreshments were provided. Providing food increased the comfort level of the participants; they were more at ease and open, and the researcher believed this gesture did not influence participation in any way. The focus group was held in a large group study rooms in the library to keep the location neutral.

The researcher provided an outline of questions; however, as is typical, the focus group did veer from the original questions as participants responded to the researcher and one another. The researcher took notes during the focus group and recorded the session with an audio recorder. The audio recording was helpful for review once the focus group had concluded. A co-moderator was also present to take additional notes and to clarify details of the discussion. After conducting the focus group, the researcher wrote additional notes and thoughts with important descriptions and episodes captured, and she constructed an account of the collective conversation using interpretive commentary [56]. These reflections helped shape questions included in the interview phases of the study and were instructive in creating initial coding themes. The focus group protocol (Appendix K) was directly informed by the survey results, although it did change somewhat from the proposed protocol based on those findings. The focus group protocol included factors identified by the SCCT as follows:

Table 5:
*SCCT Factors Addressed in Focus Group(s)*

| **Sets of Factors Identified by the SCCT** | **Addressed in Focus Group(s)** |
| --- | --- |
| Individual’s characteristics, experience, background, and interests upon entering graduate school | - Introductions with brief background - Personal experience shared - Interests throughout school years and prior to graduate school |
| Learning and performance in graduate school; skills acquired; the career search; interaction with mentors, faculty, and other students and researchers | - How they plan to search for a career - Career search sector(s) that have been encouraged or discouraged - Formal and informal statements by faculty and other students and researchers about career search, trajectory, or positions |
| Career path and necessary skills and abilities that should have been included in graduate school and postdoctoral training to aid in career development and the transition to work in or out of academia | - Careers anticipated and skills they believe may be necessary - Identifying the skills and topics they believe missing from their current career development |

The researcher also asked the participants to send her application documents they had saved, such as cover letters or emails for postdoctoral positions, personal statements to graduate school, and if still available, undergraduate school application essays or statements. Participation was voluntary and thus not mandatory or expected. Participants were encouraged to supply the researcher with photocopies of original documents, send an electronic document, or provide original documents (of which the researcher obtained photocopies and returned originals to the participant). Identifiable information was masked from the documents for participant privacy and protection. The researcher anticipated receiving only a couple of these documents to review for words and statements, which affirmed or oppose their focus group statements or the later phase interviews with the other groups.

As approval for the study was received from the IRB at Northeastern University and the research site prior to phase 1, the researcher proceeded to phase 2 with participants who met the criteria and had opted to continue with the study. At the beginning of the focus group meeting, the researcher reviewed the purpose of the study, known potential risks and benefits, and the plans for disseminating the findings from the study. Participants all signed consent forms (Appendix B) and were reminded they could withdraw or retract statements or documents during any point of the study.

### Phase 3: interviews with *trained* PhD scientists and document review.

The majority of the data was derived from qualitative interviews with the *Trained* group of PhD scientists. The interview is regarded as the most important data source in qualitative research, particularly for the case study approach [45]. Unstructured interviews were not appropriate for this study, as they are too open to address the research questions and sub-questions. Qualitative interviews were thus semi-structured to address research questions, but the researcher also allowed the process to be shaped by the interviewees, who were viewed as conversational partners elaborating and providing depth and detail to form a rich or thick description [49]. The interview protocol was edited after the focus group to address new data. The interview protocol included an introductory protocol and a few questions on the interviewees’ backgrounds; the interviewer then proceeded to ask the main open-ended interview questions about the research topic (Appendix L). The open and flexible design of a semi-structured interview or an interview guide allowed the participants to shape and introduce experiences and meaning to the topic that the researcher had not anticipated; the design permitted the researcher to probe for more information or clarification.

The structure and technique for conducting interviews was guided by Seidman [50]. Interviews were expected to last between 45 and 60 minutes; several were shorter, and two were double the expected length of time. Most interviews were conducted via telephone. Many interviewees did not reside nearby. If an interviewee was within a day’s driving distance, he/she was offered an in-person interview. If an interviewee was outside of reasonable driving distance or preferred not to meet in person, the researcher offered to conduct interviews via Google Hangout, Skype, GoToMeeting, or other web conferencing software or by telephone. Options were made available to participants based on their comfort level with conferencing applications. The audio of all interviews were recorded, with participant permission. The interview protocol was pilot tested with one person who was trained in the biomedical sciences but who was not participating in the program that is the focus of this case study.

Similar to the protocol for the focus group, the researcher took written notes during the interviews. After conducting the interview, she wrote additional notes and thoughts, with important descriptions and episodes captured, and constructed the account through interpretive commentary [56]. The audio recordings were extremely helpful in constructing the transcripts and reviews after the interview took place, and they were used as a reference for intonation and facial expressions that cannot be captured through a written transcript. Notes taken during the interview helped the researcher organize initial thoughts and themes, and to check for accuracy; they were also considered a back-up source of documentation in case the audio recording had failed.

As approval for the study was received from the IRB at Northeastern University and the research site prior to Phase 1, the researcher proceeded to Phase 3 with participants who met the criteria and who had opted to continue with the study. At the beginning of each interview, the researcher reviewed the purpose of the study, known potential risks and benefits, and the plans for disseminating the findings from the study. Participants signed consent forms (Appendix B), and were reminded they could withdraw or retract statements or documents at any point during the study.

Table 6:
*SCCT Factors Addressed in Phase 3 Interviews*

| **Sets of Factors Identified by the SCCT** | **Addressed in Interviews** |
| --- | --- |
| Individual’s characteristics, experience, background, and interests upon entering graduate school | - Introductions with brief background - Personal experience shared - Interests throughout school years and prior to graduate school |
| Learning and performance in graduate school; skills acquired; the career search; interaction with mentors, faculty, and other students and researchers | - How they plan to search for a career - Perceptions of career sector(s) and how those have been influenced - Formal and informal statements by faculty and other students and researchers about career search, trajectory, or positions - Listing career development skills acquired during training - Reflections on learning and performance during training |
| Career path and necessary skills and abilities that should have been included in graduate school and postdoctoral training to aid in career development and the transition to work in or out of academia | - Careers anticipated and skills they believe may be necessary - Identifying the skills and topics they believe missing from their current career development |

The researcher also asked the participants if they would be willing to send application documents they may have saved, such as cover letters or emails for postdoctoral positions, personal statements to graduate school, and if they were still available, undergraduate school application essays or statements. Participation was voluntary, thus not mandatory or expected. Participants were asked to supply the researcher with a photocopy of the original, send an electronic document, or the researcher obtained a photocopy and returned the original to the participant. Identifiable information was masked from the documents for participant privacy and protection. The researcher anticipated receiving only a couple of these documents, and those received were reviewed for words and statements which affirmed or opposed the focus group statements or the later phase interviews with the other groups.

### Phase 4: interviews with *faculty and administrators* and document review.

Qualitative interviews with *Faculty* and *Administrators* comprised the fourth phase of the study, though as previously mentioned, this phase ran concurrently with other phases of the study. These interviews were similar to those in Phase 3, in that they were semi-structured, flexible, and included mostly open-ended questions. The *Faculty* (Appendix M) and *Administrators* (Appendix N) interview protocols were somewhat modified throughout the study as new data was uncovered. The interview protocols included an introductory protocol and a few questions on interviewee backgrounds before the interviewer asked the main interview questions about the research topic. The protocol was tested in a mock session through which the researcher received feedback from a peer who had worked in faculty engagement and higher education administration at another institution.

Interviews lasted between 45 and 60 minutes; two were conducted in person and two were conducted over the telephone. As the faculty mentors were employed at the research site with easy access, the researcher offered in-person interviews, though participants were given the chance to elect to have a web/telephone conference interview instead. For the comfort level of the participant, the researcher provided several options for locations for meeting, such as the participant’s office or a neutral conference room or meeting space. All interviews were audio recorded, with the participants’ permission.

As with Phases 2 and 3, the researcher took notes during the interview. After conducting the interviews, the researcher wrote additional notes and thoughts with important descriptions and episodes captured, and constructed the account with interpretive commentary [56]. The recording was helpful in transcribing the interview and in reviewing after the interview took place. It also served as a reference for intonations that could not be captured through a written transcript. The notes taken during the interview assisted with initial thoughts and accuracy and also served as a backup in the case that the recording failed.

As approval for the study was received from the IRB at Northeastern University and the research site prior to Phase 1, no additional approval for this phase was necessary. At the beginning of each interview, the researcher reviewed the purpose of the study, known potential risks and benefits, and the plans for disseminating the findings from the study. Participants provided verbal recorded consent and were reminded they could withdraw or retract statements or documents at any point during the study.

Table 7*:
SCCT Factors Addressed in Phase 4 Interviews*

| **Sets of Factors Identified by the SCCT** | **Addressed in Interviews** |
| --- | --- |
| Individual’s characteristics, experience, background, and interests upon entering graduate school | - Perception of trainees’ interests upon entering training and any changes during training |
| Learning and performance in graduate school; skills acquired; the career search; interaction with mentors, faculty, and other students and researchers | - Interactions with trainees about career search, trajectory, positions - Perceptions of career sector(s) - Listing career development skills covered during training |
| Career path and necessary skills and abilities that should have been included in graduate school and postdoctoral training to aid in career development and the transition to work in or out of academia | - Careers anticipated and skills they believe may be necessary - Identifying the skills and topics they believe taught and missing from current career development |

The researcher asked the participants if they would be willing to send documents or PowerPoint presentations they may have saved regarding career development skills. Participation was voluntary, and thus not mandatory or expected. Participants were asked to supply the researcher with a photocopy of the original, send an electronic document, or the researcher obtained a photocopy and returned the original to the participant. Identifiable information was masked from the documents for participant privacy and protection.

## Data Management

To minimize risks to individuals and their organizations, names and identifying information have been hidden from the findings and analysis. A pseudonym was used for each participant with an identity key stored securely in a password-protected file kept separately from the transcript files and recordings. Documents collected also had names and identifying information erased or replaced with pseudonyms. Electronic files were backed up through an online secure data storage system under two layers of password protection. The computer and online data storage was, throughout the entire process, only accessible to the researcher. Physical documents and consent forms were stored in a locked box with the key location known only by the researcher. The policies of both the federal government and Northeastern University require data destruction by shredding after three years, which will appropriately take place.

Stake and Savolainen [56] recommend displaying the progress of the study through a data storage system with a calendar, list of phone numbers, observation notes, list of expenses, etc. A master list of data sources has been maintained so that the researcher can easily retrieve the information. Many recommend that researchers keep a journal to reflect on the research process, revelations, shifts in thinking and understanding, and interpretive decisions [45, 61-63]. In addition to using multiple sources of evidence, Yin [45] presented two other principles of data collection: creating a case study database and maintaining a chain of evidence to increase reliability. The use of a reflective journal was instrumental in aiding the researcher in data management; it acted as an audit trail, and upheld decisions the researcher made.

## Data Analysis

Research through the case study methodology is distinctive in explaining, describing, benefitting, illustrating, and exploring situations. The primary task of conducting research, according to Stake and Savolainen [56], is to understand the case, discover relationships, probe issues, comprehend collective and categorical data, lessen the burden of clarifying descriptions, and make sophisticated interpretations accessible to readers through thick descriptive constructivism in a final report. The responsibility of the researcher to make the findings accessible means considerable focus must be placed on the data analysis process. Analyzing data and patterns does not begin near the end of the project but is ongoing because the interpretive researcher engages in observation, renewed inquiry, and explanation through a progressive focusing: “triangulation regularly sends us back to the drawing board” [56, pp. 114). This section describes the data analysis process.

### Close-ended survey questions.

The survey included close-ended and open-ended questions. The close-ended survey data was viewed within the Google Form data visualization tools and was also exported as a spreadsheet to allow the researcher to calculate descriptive statistics, look for outliers, and search for patterns and key words or phrases. The close-ended questions allowed for the use of pie or bar charts to demonstrate findings. A summary of data was analyzed and reported as descriptive statistics. Though this study includes some quantitative data, it is predominately a qualitative study and has been analyzed as such.

### Open-ended survey questions, focus group, interviews, and documents.

Open-ended survey responses and documents were loaded into MAXQDA, a software package that aids the researcher in analyzing qualitative data. Responses from the focus groups and interviews were transcribed word for word by an external service, Rev.com, and the researcher reviewed all transcripts for accuracy. The transcripts were provided to the participants for review as a method of member-checking. Finally, the focus group and interview transcripts were uploaded into MAXQDA to code along with the open-ended survey responses and collected documents using Saldaña [47] as a guide.

All responses to data collection methods were read through in their entirety at least twice to gain a sense of broad themes. Documents (for this section, documents refers to documents supplied to the researcher, as well as documents compiling survey responses and transcripts from the focus groups and interviews) were then analyzed individually before conducting a cross-analysis with the rest of the documents to discover overarching themes and identify outliers. The researcher continued to be reflective throughout the data collection and analysis process. An inductive approach allows researchers to let themes and patterns emerge from the raw data with less bias, thus the researcher used an inductive approach to analyze and make sense of many data. Analysis often reveals results in words, pictures, diagrams, and thematic mapping, and allows the researcher to highlight important direct quotations.

In first cycle coding, each focus group transcript, interview transcript, document, and documented survey responses were reviewed in isolation using MAXQDA, a qualitative software package with easily viewable display. This helped the researcher create an in-depth line-by-line analysis and an initial open coding of data. The researcher continued taking notes and writing journal reflections regarding lines of thinking, potential themes or patterns, and implicit meanings behind intonation and facial expressions. The researcher wrote descriptive summaries to capture the essence of what the participants had said.

Initial codes were consolidated into more general thematic codes in iterative patterns during the second level of coding. These themes created broad categories and condensed the data into more manageable patterns to allow the researcher to begin understanding themes across transcripts and to analyze findings. Predetermined codes from the theoretical framework chosen, the SCCT, helped the researcher to further organize and analyze data, but these were only used after the first and second coding cycles.

The next step was axial coding and cross analysis of documents for emergent themes and patterns. Themes, categories, and patterns emerging from individual document analysis were reviewed for similarities and differences across all documents. A word table was created to display data from individual documents and among all documents. Code landscaping and mapping were used to highlight themes and conclusions across data sources. Finally, provisional coding was used with categories and themes from the literature review and the SCCT. Provisional codes were only be used after the first two levels of coding to prevent the findings from being distorted by preconceptions. These levels of coding are organized in systematic ways so as to compare data sources that increase the validity of analysis. Findings have taken the form of narratives because of the nature of qualitative data, but they also include tables and figures.

## Trustworthiness

The study sought to uphold trustworthiness and verification with limited bias. As previously noted, it was unlikely that the study would have caused harm to participants, and the study was approved by the IRB for the protection of human participants prior to contacting potential participants. The researcher maximized the confidentiality of the participants, collected data through safe and comfortable methods, and was mindful of any power imbalance or bias. The statements of the researcher’s bias and the purpose and lengths the researcher has taken during the study design to each potential participant indicate how the researcher has intended to minimize potential threats to internal validity.

For representativeness, the researcher surveyed all past trainees, interviewed two individuals in each of the non-academic biomedical science career sectors, conducted a focus groups with current trainees, interviewed faculty mentors and administrators, and reviewed documents used in graduate school applications, career searches, and career development. The multiple types of participants and data collection methods were used for representativeness and triangulation of data. Outliers, surprising, or conflicting evidence have not been eliminated from the findings.

Member-checking was facilitated by providing participants with a copy of the transcript to review for any edits, clarifications, issues, or omissions. Feedback and clarification were used to increase the authenticity of the representation of the participants’ views. External validity was sought with fully described participants, and settings, processes. The ability (or inability) to generalize findings to other settings or contexts has been acknowledged. Additionally, for external validity, the study design was evaluated by non-participants for clarity and question congruence.

Terms have been defined within the methodology section of the dissertation and within the research questions. The researcher sought validity of the instrumentation by asking the same questions within each phase of the study and by using the same analysis procedure to analyze the data from each phase. The researcher sought to use rich and meaningful descriptions. The researcher has discussed whether the findings are transferable and applicable to other populations and settings. Emerging patterns have been used to code data, rather than deductive coding methods to also lessen bias and allow the data to speak for itself [49].

## Limitations

The case study portrays multiple views of the case, and it has been the responsibility of the researcher to assist the readers to gain a high quality understanding of the issue [56]. Though the study encompasses multiple sample pools and data collection methods, and the researcher has detailed the research design, analysis, and measures to increase trustworthiness, the study is not without its limitations.

The main limitation is generalizability of the findings. Participants were recruited from a specific pool that had participated in a specific program at one highly selective institution. Their experiences as written in the findings must not be understood as representative of all biomedical PhD scientists. Yin [45] regarded case studies as significant if they are either unusual but have general public interest or if the underlying issues are nationally important. This case study fulfills the latter criterion. Though the study population and sample may not be generalizable, the literature review demonstrated that similar issues are found at institutions throughout the United States and around the world. Though the findings from this study may not be exactly transferable to other populations and settings, there may be overlapping aspects, which can inform and benefit other populations.

Additionally, the number of factors incorporated in SCCT encompass more roles and intersections than can be discussed and understood. Though the researcher has taken lengths to inquire about the many factors that SCCT accounts for, there may be factors, which have been overlooked or are not yet discovered. SCCT considers individual characteristics and experiences, interactions with others, and external influences, which is a scope larger than any one research study can discover and explain.

# Appendix A

Permission Request for Access to Study Contacts


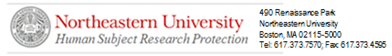


2/22/16
[name and address of training program director]

RE: Permission to Conduct Research Study

Dear [director name]:

As you know, I am currently enrolled in the Doctor of Education in Higher Education Administration program at Northeastern University in Boston, MA, and I am in the process of writing my dissertation.  The study is entitled *Navigating the Path to a Biomedical Science Career*. I am writing to request permission and access to your program’s contacts.

The central research question driving this case study is: How does [a southeastern tier 1 research university] train and support its biomedical PhD scientists for navigating their career paths? Data collected will illuminate the how the process influences the outcome of the job search and explore other factors and experiences contributing to career paths sought and chosen. A practical sub-goal of this study is to uncover suggestions and ideas about skills that should be cultivated to benefit and prepare biomedical PhD scientists for employment after graduation.

If approval is granted, current and former trainees will be invited to complete an online survey. The survey process should take no longer than 15 minutes.  Participants may self-select into the next phase of the study, which includes participation in a focus group or an interview, depending on training or career stage. Also, current faculty mentors of the training program will be invited to participate in an in-person or web conference interview. The focus groups and interviews will take no longer than one hour each. Individual results of this study will remain absolutely confidential and anonymous.  Administrators of the departments, other NIH training grants, the Office of Postdoctoral Affairs, and the Graduate Programs Office may be included in the interview phase of the study to learn of the training process and opportunities available outside of the research laboratory for graduate students and postdoctoral fellows. No costs will be incurred by either your school/center or the individual participants.

Your approval to conduct this study will be greatly appreciated.  I will follow up with a telephone call next week and would be happy to answer any questions or concerns that you may have at that time. You may contact me at my email address: zimmerman.an@husky.neu.edu.

If you agree, kindly return the signed form.  Alternatively, kindly submit a signed letter of permission on your institution’s letterhead acknowledging your consent and permission for me to conduct this study with your program’s contacts.

Sincerely,

Andrea Zimmerman
Doctoral Student, Northeastern University

cc:        Dr. Kelly Conn, Research Advisor, Northeastern University

Approved by:

_______________________________         ______________________________       _________

Print your name and title here     Signature                                Date

# Appendix B

Signed Consent Form


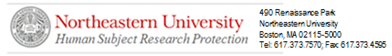


Title of Project: Navigating the Path to a Biomedical Science Career

Name of Investigators: Andrea McNeely Zimmerman (Student Researcher)

Northeastern University, Department of Education—Higher Education Administration concentration

Study Contact: 434-214-0394 or zimmerman.an@husky.neu.edu.

Faculty Advisor: Kelly Conn, PhD ([k.conn@neu.edu](mailto:k.conn@neu.edu))

Informed Consent to Participate in a Research Study

We are inviting you to take part in a research study. This form will tell you about the study, but the researcher will explain it to you first. You may ask this person any questions that you have. When you are ready to make a decision, you may tell the researcher if you want to participate or not. You do not have to participate if you do not want to. If you decide to participate, the researcher will ask you to sign this statement and will give you a copy to keep.

Why am I being asked to take part in this research study?

I am asking you to participate in this study because you:

1. have been supported for some amount of time by the [training program name] while in training at [institution name]
2. are a faculty mentor of the [training program name] at the [institution name]
3. are an administrator of programs related to biomedical sciences at [institution name]

Why is this research study being done?

This purpose of this study is to understand the process by which biomedical PhD scientists are trained and supported for navigating a future career path.

What will I be asked to do?

If you decide to take part in this study, we will ask you to answer open-ended questions in either a focus group or one-on-one interview about career development opportunities, perceptions, and preparation. You will be asked to also share documents, which may include graduate school personal statements, postdoctoral position cover letters, notes or slides from career development sessions, or other relevant documents.

Where will this take place and how much of my time will it take?

For those taking part in the focus group, participants will meet in a library group study room or neutral conference room or meeting space. The focus groups will last approximately one hour and will be audio recorded with the participants’ permission.

For the comfort level of the interviewee, the researcher will offer several locations for meeting, such as the participant’s office or a neutral conference room or meeting space. Interviews may also be offered as web or telephone conferences for participant comfort and convenience. All interviews will be audio recorded, with the participants’ permission.

Will there be any risk or discomfort to me?

The foreseeable risks or discomforts of the study are minimal; however, you may feel a little uncomfortable answering personal questions.

Will I benefit by being in this research?

There are no direct benefits to you from participating in this study. However, your responses may help us learn more about and strengthen the career development support efforts and opportunities for biomedical scientists.

Who will see the information about me?

Your part in this study will be handled in a confidential manner. Any reports or publications based on this research will use only group data and will not identify you or any individual as being affiliated with this project. Data will be stored in a double password protected server without identifying information.

If you have any questions regarding electronic privacy, please feel free to contact Mark Nardone, NU’s Director of Information Security via phone at 617-373-7901, or via email at [privacy@neu.edu](mailto:privacy@neu.edu).

If I do not want to take part in the study, what choices do I have?

The decision to participate in this research project is voluntary. You do not have to participate.

What will happen if I suffer any harm from this research?

No special arrangements will be made for compensation or for payment for treatment solely because of my participation in this research.

Can I stop my participation in this study?

Yes. You can refuse to answer any question. Even if you begin the focus group or interview, you can stop at any time. At the end, you will be offered an opportunity to review the focus group or interview transcript for clarifications and omissions.

Who can I contact if I have questions or problems?

If you have any questions about this study, please feel free to contact Andrea Zimmerman, the person mainly responsible for the research, via phone at 434-214-0394 or email at zimmerman.an@husky.neu.edu. You can also contact Kelly Conn via email at [k.conn@neu.edu](mailto:k.conn@neu.edu).

Who can I contact about my rights as a participant?

If you have any questions regarding your rights as a research participant, please contact Nan C. Regina, Director, Human Subject Research Protection, 490 Renaissance Park, Northeastern University, Boston, MA 02115. Tel: 617.373.4588, Email: n.regina@neu.edu. You may call anonymously if you wish.

Will I be paid for my participation?

You will not be paid for your participation in this study.

Will it cost me anything to participate?

No.

Is there anything else I need to know?

**This study has been reviewed and approved by the Northeastern University Institutional Review Board (# xx-xx-xx).** [protocol # will be provided to you by the HSRP office].

I agree to take part in this research.

| **Signature of person agreeing to take part** | **Date** |
| --- | --- |
| **Printed name of person above** |  |
| **Signature of person who explained the study to the participant above and obtained consent** | **Date** |
| **Printed name of person above** |  |

# Appendix C

The Unsigned Informed Consent Email for Web-Based Online Surveys


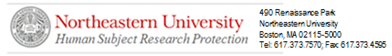


Title of Project: Navigating the Path to a Biomedical Science Career

Name of Investigators: Andrea McNeely Zimmerman (Student Researcher)

Northeastern University, Department of: Education—Higher Education Administration concentration

Faculty Advisor: Kelly Conn, PhD (k.conn@neu.edu)

Study Contact: 434-214-0394 or zimmerman.an@husky.neu.edu.

Subject Line: Request to participate in study: navigating a biomedical science career path

**Request to Participate in Research**

Dear ___________,

My name is Andrea McNeely Zimmerman. I am a student in the Doctor of Education program at Northeastern University and am currently conducting a study for my dissertation and am seeking research participants.

I would like to invite you to participate in a web-based online survey. This survey is part of a resource study, the purpose of which is to understand the process by which biomedical PhD scientists are trained and supported for navigating a future career path. This survey should take about 12-15 minutes to complete.

I am asking you to participate in this study because you have been supported for some amount of time by the [training program name] while in training at the [university name].

**The decision to participate in this research project is voluntary.** You do not have to participate, and you can refuse to answer any question. Even if you begin the web-based online survey, you can stop at any time. At the end of the survey, you may elect to participate in a more in-depth phase of the study through either a focus group or an interview.

**The possible risks or discomforts of the study are minimal**; however, you may feel a little uncomfortable answering personal survey questions.

**There are no direct benefits to you from participating in this study.** However, your responses may help us learn more about and strengthen the career development support efforts and opportunities for biomedical scientists.

**You will not be paid for your participation in this study.**

**Your part in this study will be handled in a confidential manner. Any reports or publications based on this research will use only group data and will not identify you or any individual as being affiliated with this project.** Data will be stored in a double password protected server without identifying information.

**If you have any questions regarding electronic privacy**, please feel free to contact Mark Nardone, NU’s Director of Information Security via phone at 617-373-7901, or via email at [privacy@neu.edu](mailto:privacy@neu.edu).

**If you have any questions about this study**, please feel free to contact Andrea Zimmerman, the person mainly responsible for the research, via phone at 434-214-0394 or email at zimmerman.an@husky.neu.edu. You can also contact Kelly Conn via email at [k.conn@neu.edu](mailto:k.conn@neu.edu).

**If you have any questions regarding your rights as a research participan**t, please contact Nan C. Regina, Director, Human Subject Research Protection, 490 Renaissance Park, Northeastern University, Boston, MA 02115. Tel: 617.373.4588, Email: n.regina@neu.edu. You may call anonymously if you wish.

**This study has been reviewed and approved by the Northeastern University Institutional Review Board (# xx-xx-xx).** [protocol # will be provided to you by the HSRP office].

**By clicking on the survey link below [Or the “accept” button below] you are indicating that you consent to participate in this study. Please print out a copy of this consent form for your records.**

<http://_____________________________________________________>

Please fill out the survey by MM/DD/YYYY (2 weeks from send date).

Thank you for your time.

Andrea Zimmerman

# Appendix D

Thank You Message Appearing After Survey Completion

Dear ___________________,

Thank you for completing the survey portion of the study. Your insight and input is greatly appreciated and will add significantly to this study. If you have agreed to participate in the next phase of the research study, you will receive an email or phone call to schedule your participation.

Thank you again for your willingness to participate in this study.

Sincerely,

Andrea Zimmerman

# Appendix E

Reminder Email

Subject Line: Request to participate in study: navigating a biomedical science career path

Dear ___________,

One week ago you received an email (below) about a research study that I am doing for my doctoral dissertation.

This is a reminder to complete the survey by MM/DD/YYYY, if you are interested in participating: <http://_____________________________________________________>

Thank you again for considering participation in the study.

Regards,

Andrea Zimmerman

******Note****: This is a follow up email that will be sent to All Trainees that do not respond within seven days of the initial email. For the follow up email, the initial email will be forwarded to students so they can easily view the information included and respond appropriately.*

# Appendix F

Recruitment Email for Focus Group Phase

Subject: Thank you for participating. Scheduling request for next study phase.

Dear ___________________,

Thank you for completing the survey portion of the study. Your insight and input is greatly appreciated and will add significantly to this study. You are receiving this email as you have agreed to participate in the next phase of the research study.

You have been selected for the focus group phase. I would like to schedule a time at the convenience of participants. **Please let me know whether a morning, afternoon, evening, or weekend time is best.** We will meet in a neutral space in one of the library’s group meeting rooms. The focus group will last approximately one hour.

If you have any questions or concerns, please stop by my office (McKim G144), call 434-214-0394, or email Zimmerman.an@husky.neu.edu. Thank you again for your willingness to participate in this study. I look forward to speaking with you.

Sincerely,

Andrea Zimmerman

# Appendix G

Recruitment Email for *Trained* Interviews

Subject: Thank you for participating. Scheduling request for next study phase.

Dear ___________________,

Thank you for completing the survey portion of the study. Your insight and input is greatly appreciated and will add significantly to this study. You are receiving this email as you have agreed to participate in the next phase of the research study.

You have been selected for the interview phase. I would like to schedule a time at your convenience. If you are within a day’s driving distance of [research site], we could hold the interview in your town at a location convenient and comfortable for you. Regardless of location, the interview can be held over web conferencing or telephone. The interview will last approximately 45 minutes. **Please let me know which option is most convenient and whether a morning, afternoon, evening, or weekend time is best.**

If you have any questions or concerns, please call 434-214-0394 or email Zimmerman.an@husky.neu.edu. Thank you again for your willingness to participate in this study. I look forward to speaking with you.

Sincerely,

Andrea Zimmerman

# Appendix H

The Unsigned Informed Consent Email for Interviews of *Faculty* Mentors


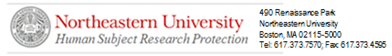


Title of Project: Navigating the Path to a Biomedical Science Career

Name of Investigators: Andrea McNeely Zimmerman (Student Researcher)

Northeastern University, Department of Education—Higher Education Administration concentration

Study Contact: 434-214-0394 or zimmerman.an@husky.neu.edu.

Faculty Advisor: Kelly Conn, PhD (k.conn@neu.edu)

Subject Line: Request to participate in study: Navigating a Biomedical Science Career Path

**Request to Participate in Research**

Dear ___________, (will use the mail merge function to automatically fill in names)

My name is Andrea McNeely Zimmerman. I am a student in the Doctor of Education program at Northeastern University and am currently conducting a study for my dissertation and am seeking research participants. The purpose of this study is to understand the process by which biomedical PhD scientists are trained and supported for navigating a future career path. I am asking you to participate in this study because you are one of the mentors for the [training program name] at the [university name].

I would like to invite you to participate through an in-person, web conferencing, or telephone interview. The interview will take approximately 30 minutes. If you decide to take part in this study, we will ask you to answer open-ended questions in a one-on-one interview about career development opportunities, perceptions, and preparation. You will be asked to also share documents, which may include graduate school personal statements, postdoctoral position cover letters, notes or slides from career development sessions, or other relevant documents.

**The possible risks or discomforts of the study are minimal.** You may feel a little uncomfortable answering personal questions.

**There are no direct benefits to you from participating in this study.** However, your responses may help us learn more about and strengthen the career development support efforts and opportunities for biomedical scientists.

**Your part in this study will be handled in a confidential manner.** Any reports or publications based on this research will use only group data and will not identify you or any individual as being affiliated with this project. Data will be stored in a double password protected server without identifying information.

**The decision to participate in this research project is up to you.** You do not have to participate and you can refuse to answer any question. Even if you begin the interview, you can stop at any time. At the end, you will be offered an opportunity to review the interview transcript for clarifications and omissions.

**You will not be paid for your participation in this study.**

**If you have any questions about this study,** please feel free to contact Andrea Zimmerman, the person mainly responsible for the research, via phone at 434-214-0394 or email at zimmerman.an@husky.neu.edu. You can also contact Kelly Conn via email at [k.conn@neu.edu](mailto:k.conn@neu.edu).

**If you have any questions regarding your rights in this research**, please contact Nan C. Regina, Director, Human Subject Research Protection, 490 Renaissance Park, Northeastern University, Boston, MA 02115. Tel: 617.373.4588, Email: n.regina@neu.edu. You may call anonymously if you wish.

**If you are willing to participate in the interview phase of the study, please respond with three dates and times you are available during the next two weeks. Also, let me know whether you prefer an in person, telephone, or web conference setting for your interview.**

Thank you for your time.

Andrea Zimmerman

# Appendix I

The Unsigned Informed Consent Email for Interviews of *Administrators*


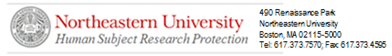


Title of Project: Navigating the Path to a Biomedical Science Career

Name of Investigators: Andrea McNeely Zimmerman (Student Researcher)

Northeastern University, Department of: Education—Higher Education Administration concentration

Faculty Advisor: Kelly Conn, PhD (k.conn@neu.edu)

Study Contact: 434-214-0394 or zimmerman.an@husky.neu.edu.

Subject Line: Request to participate in study: Navigating a Biomedical Science Career Path

**Request to Participate in Research**

Dear ___________, (will use the mail merge function to automatically fill in names)

My name is Andrea McNeely Zimmerman. I am a student in the Doctor of Education program at Northeastern University and am currently conducting a study for my dissertation and am seeking research participants. This purpose of this study is to understand the process by which biomedical PhD scientists are trained and supported for navigating a future career path. I am asking you to participate in this study because you are one of the administrators for programs at [university name].

I would like to invite you to participate through an in person, web conferencing, or telephone interview. The interview will take approximately 45 minutes. If you decide to take part in this study, we will ask you to answer open-ended questions in a one-on-one interview about career development opportunities, perceptions, and preparation.

**The possible risks or discomforts of the study are minimal**.

**There are no direct benefits to you from participating in this study.** However, your responses may help us learn more about and strengthen the career development support efforts and opportunities for biomedical scientists.

**Your part in this study will be handled in a confidential manner.** Any reports or publications based on this research will use only group data and will not identify you or any individual as being affiliated with this project. Data will be stored in a double password protected server without identifying information.

**The decision to participate in this research project is up to you.** You do not have to participate and you can refuse to answer any question. Even if you begin the study, you may withdraw at any time. You will also have the opportunity to review the interview transcript.

**You will not be paid for your participation in this study.**

**If you have any questions about this study**, please feel free to contact Andrea Zimmerman, the person mainly responsible for the research, via phone at 434-214-0394 or email at zimmerman.an@husky.neu.edu. You can also contact Kelly Conn via email at [k.conn@neu.edu](mailto:k.conn@neu.edu).

**If you have any questions about your rights in this research**, please contact Nan C. Regina, Director, Human Subject Research Protection, 490 Renaissance Park, Northeastern University, Boston, MA 02115. Tel: 617.373.4588, Email: n.regina@neu.edu. You may call anonymously if you wish.

**If you are willing to participate in the interview phase of the study, please respond with three dates and times you are available during the next two weeks. Also, let me know whether you prefer an in person, telephone, or web conference setting for your interview.**

Thank you for your time.

Andrea Zimmerman

# Appendix J

Survey Instrument

**Age (fill-in)**

**Gender**

**Years in training at [UNIVERSITY] (YYYY-YYYY) _________ - __________**

**Trained at [UNIVERSITY] as**

- **predoc**
- **postdoc**

**What is your current career sector?**

- **Academic**
- **Clinical/healthcare**
- **Government**
- **Industry**
- **Non-research (e.g. consulting, scientific writing, or unrelated area)**

**If you have worked in multiple career sectors, please mark those prior to your current sector.**

- **Academic**
- **Clinical/healthcare**
- **Government**
- **Industry**
- **Non-research (e.g. consulting, scientific writing, or unrelated area)**

**Which career sector were you interested in prior to graduate school?**

- **Academic**
- **Clinical/healthcare**
- **Government**
- **Industry**
- **Non-research (e.g. consulting, scientific writing, or unrelated area)**

**On a scale of 1-10, with 1 being not at all favorable and 10 being most favorable, please rate your mentor’s encouragement of the following career sectors:**

**Academic 1 2 3 4 5 6 7 8 9 10**

**Clinical/healthcare 1 2 3 4 5 6 7 8 9 10**

**Government 1 2 3 4 5 6 7 8 9 10**

**Industry 1 2 3 4 5 6 7 8 9 10**

**Non-research (e.g. consulting, 1 2 3 4 5 6 7 8 9 10**

**scientific writing, or unrelated area)**

**On a scale of 1-10, with 1 being not at all favorable and 10 being most favorable, please rate the interactions with your mentor during training at [UNIVERSITY].**

**1 2 3 4 5 6 7 8 9 10**

**On a scale of 1-10, with 1 being not at all favorable and 10 being most favorable, please rate the interactions with your mentor after training at [UNIVERSITY].**

**1 2 3 4 5 6 7 8 9 10**

**On a scale of 1-10, with 1 being not at all favorable and 10 being most favorable, please rate the career development opportunities you had while in training at [UNIVERSITY].**

**1 2 3 4 5 6 7 8 9 10**

**On a scale of 1-10, with 1 being not at all prepared and 10 being most prepared, please rate how prepared you felt for your career search post-training.**

**1 2 3 4 5 6 7 8 9 10**

**On a scale of 1-10, with 1 being not at all favorable and 10 being most favorable, please rate how prepared you felt for your career(s) post-training.**

1. **2 3 4 5 6 7 8 9 10**

**Other than bench or technical skills, which skills are needed in your current position? ______________________________________________________________________________**

**Did you receive these skills during training at [UNIVERSITY]? Yes No**

**Would you be willing to participate in a focus group or interview? If so, please include your preferred contact information to use in scheduling further participation.**

**First name ______________________________________________________________**

**Telephone Number _______________________________________________________**

**Email Address __________________________________________________________**

**Thank you for your time and willingness to participate in this survey. If you are selected to participate in one of the next phases of the study, you will be contacted within 2 months.**

# Appendix K

Focus Group Protocol

Good afternoon/evening. Thank you for taking the time to join our discussion of the training environment at [university name].

My name is Andrea Zimmerman, and this is __________________, who will act as an impartial assistant moderator. As stated in our previous communication, I am conducting research on how biomedical scientists navigate career paths. In particular, I hope to learn how PhD scientists could be better supported and trained for careers post-training.

We want to talk with you about your experiences as graduate students and postdoctoral fellows so we’ll be asking about what originally drew you to biomedical science and this program, and what it has been like for you here since you began.

Before we begin, let me suggest some things to make our discussion more productive. Because we will be recording for an accurate record, it is important that you speak up and that you only speak one at a time. We don’t want to miss any of your comments. We’ll only use first names here. No reports will link what you say to your name, department, or institution. In this way, we will maintain your confidentiality. In addition, we ask that you also respect the confidentiality of everyone here. Please do not repeat what others said when you leave this room.

During this time, I will ask you questions, and I will listen to what you have to say. I will not participate in the discussion so please feel free to respond to each other and to speak directly to others in the group.

We want to hear from all of you. We are interested in all viewpoints, common and uncommon experiences, so I may sometimes encourage someone who has been quiet to talk, or by asking someone to hold off for a few minutes.

If it is OK with you, we will turn on the recorder and start now.

This student focus group is being conducted for the Biomedical Science Career Path Study on MM/DD/YYYY by Andrea Zimmerman and assistant moderator.

Our start time is HH:MM.

I. Let’s begin with introductions.

A. Please tell us your first name, what program or area of training you’re in, and whether you are a grad student or postdoc.

II. Now that we know a little about you, I would like you to think back to when you first decided you wanted to be a scientist.

B. What was it that drew you to science and biomedical science in particular?

C. What drew you to this particular program at [UNIVERSITY]?

SUMMARIZE: It looks like there were (quite a few/some) positive features of *biomedical science* and this program that motivated your initial choices. These features included: NAME CATEGORIES.

III. Now, we’d like to talk about what has happened since you entered grad school. What experiences have you had that encouraged you to continue or increased your initial enthusiasm for *biomedical science*?

D. I’d like to list any experiences you have had that have discouraged you or reduced your initial enthusiasm.

IV. We are interested in your views of career sectors. We have identified four career sectors of academic, industry, government, and non-research (for example, consulting, scientific writing, or healthcare).

E. What type of career did you intend to pursue when you were young?

F. What about in college?

G. How about now?

H. What are pros and cons of an academic career?

I. What are pros and cons of non-academic careers?

J. How did you determine these? Who in particular has influenced your views on career paths?

V. How do you feel/what do you anticipate as you look ahead to your career?

K. How prepared do you feel for the career search?

L. How prepared do you feel for your desired career sector?

M. What skills, other than bench or technical, do you anticipate needing for your career?

N. How confident are you that you have the necessary skills for your career?

O. Where did you receive training or support in these skills?

P. What changes would you recommend for career development training at [UNIVERSITY]?

VI. To summarize what we discussed, you said…

SUMMARIZE THE POSITIVE AND NEGATIVE ASPECTS, ACKNOWLEDGING

DIFFERENT POINTS OF VIEW.

VII. Does that capture the essence of what was said here? Are there any last comments?

VII. Finally, as I told you at the beginning, the purpose of this study is to get information about career paths and career development training and support in biomedical sciences at [UNIVERSITY]. As part of my analysis, I am collecting documents for review, such as personal statements for college and grad school discussing your interests and intentions, cover letters for postdoc positions, notes from career development seminars, emails from mentors, or other documents. If you have any documents you would be willing to share or if you have any thoughts after today’s focus group, please feel free to email me.

Thank you so much for your participation in the survey and focus group. Your input has been valuable for my study.

# Appendix L

*Trained* Group Interview Protocol

Information on the signed consent form (Appendix B) will be reviewed by the researcher. The interviewee will sign the consent form before the researcher continues.

1. Tell me about what drew you to biomedical science.
   1. When did you first become interested in it?
2. Tell me about your graduate and post-graduate training experience.
   1. What was your relationship with your mentor/advisor and other faculty?
3. Tell me about the climate and conversations or perceptions about career paths or sectors.
4. Who has influenced your career choice or sector choice?
5. What was your job/career search experience like?
6. Tell me about the career development opportunities that were:
   1. Available to you.
   2. Taken by you.
7. Looking back, aside from technical skills, how well prepared were you for a career?
8. What skills or topics were missing in your career development opportunities or which should remain?

# Appendix M

Faculty Interview Protocol

Information on the signed consent form (Appendix B) will be reviewed by the researcher. The interviewee will sign the consent form before the researcher continues.

1. Tell me about trainees’ interest prior to and during training.
2. Have there been shifts in career sector intention or focus?
3. What do you believe influences their career perceptions or trajectory?
4. What is your perception of each career sector? Are any favored or discouraged?
5. Which skills are needed for a career after training?
6. Which skills, other than technical, did/do you cultivate during their training?
   1. What type of career development opportunities do your trainees have (could be other programs, emails you forwarded of campus activities, workshops away, etc.)
7. Are the skills needed for different career sectors different?
8. Which skills and career development opportunities are missing and necessary for success?

# Appendix N

Administrators Interview Protocol

Information on the signed consent form (Appendix B) will be reviewed by the researcher. The interviewee will sign the consent form before the researcher continues.

1. What is your perception of each career sector? Are any favored or discouraged?
2. Which skills are needed for a career after training?
3. Which skills, other than technical, did/do you cultivate during their training?
   1. What type of career development opportunities do your trainees have (could be other programs, emails you forwarded of campus activities, workshops away, etc.)
4. Are the skills needed for different career sectors different?
5. Which skills and career development opportunities are missing and necessary for success?
6. How are topics for career development determined?
7. What kind of feedback have you received from opportunities, or what kinds of opportunities have trainees asked for?
8. Who typically provides lectures or workshops in career development skills?
   1. Broader outside of academe?

# References

1. Fuhrmann CN, Halme DG, O’Sullivan PS, Lindstaedt B. Improving graduate education to support a branching career pipeline: Recommendations based on a survey of doctoral students in the basic biomedical sciences. CBE-Life Sci Educ. 2011;10(3): 239–249. PMCID: PMC3164563
2. Fiske P. What is a PhD really worth? Nature. 2011;472(7343): 381–381.
3. Altschuld RA. US science education: The view from a practicing scientist. Rev Policy Res. 2003;20(4): 635–645.
4. Levitt DG. Careers of an elite cohort of U.S. basic life science postdoctoral fellows and the influence of their mentor’s citation record. BMC Med Educ. 2010;10(1): 80–86. PMCID: PMC2996387
5. Alberts B, Kirschner MW, Tilghman S, Varmus H. Rescuing US biomedical research from its systemic flaws. PNAS. Apr 14, 2014;111(16): 5773–5777. PMCID: PMC4000813
6. Benderly BL. Not your father’s postdoc. Science. 2005;308(5722): 717–718. PMID: 15860631
7. Benderly BL. Support for tenure-track jobs in biomedical sciences. Science. 2009;324(5923): 27. PMID: 19342563
8. Cyranoski D, Gilbert, N, Ledford H, Nayar A, Yahia, M. Education: The PhD factory. Nature. 2011;472(7343): 276–279. PMID: 21512548
9. Goldman E. NIH grantees: Where have all the young ones gone? Science. 2002;298(5591): 40–41.
10. Taylor M. Reform the PhD system or close it down. Nature. 2011;472(7343): 261. PMID: 21512530
11. Teitelbaum MS. Structural disequilibria in biomedical research. Science, 2008;321(5889): 644–645. PMID: 18669847
12. Daniels R. A generation at risk: Young investigators and the future of the biomedical workforce. PNAS. 2014;112(2): 313–318. PMCID: PMC4299207
13. Omenn GS. Grand challenges and great opportunities in science, technology, and public policy. Science. 2006;314(5806): 1696–1704.
14. Gaughan M, Robin S. National science training policy and early scientific careers in France and the United States. Res Policy. 2004;33(4): 569–581.
15. Lee H, Miozzo, M, Laredo P. Career patterns and competences of PhDs in science and engineering in the knowledge economy: The case of graduates from a UK research-based university. Res Policy. 2010;39(7): 869–881.
16. Mangematin V. PhD job market: Professional trajectories and incentives during the PhD. Res Policy. 2000;29(6): 741–756.
17. Wendler C, Bridgeman B, Cline F, Millett C, Rock J, Bell N, et al. The path forward: The future of graduate education in the United States. Princeton: Educational Testing Service; 2010.
18. Agarwal R, Ohyama A. Industry or academia, basic or applied? Career choices and earnings trajectories of scientists. Manag Sci. 2012;59(4): 950–970.
19. Basil MD, Basil DZ. The marketing market: A study of PhD supply, demand, hiring institutions, and job candidates. J Bus Res. 2006;59(4): 516–523.
20. Coggeshall PE, Norvell JC. Bogorad L, Bock RM. Changing postdoctoral career patterns for biomedical scientists. Science. 1978;202(4367): 487–493. PMID: 705338
21. Fritsch M, Krabel S. Ready to leave the ivory tower? Academic scientists’ appeal to work in the private sector. J Technol Transfer. 2012;37(3): 271–296.
22. Martinson B C, Crain AL, Anderson, MS, De Vries R. Institutions’ expectations for researchers’ self-funding, federal grant holding and private industry involvement: Manifold drivers of self-interest and researcher behavior. Academic Medicine: J Assoc Am Med Coll. 2009;84(11): 1491–1499. PMCID: PMC3071700
23. Matthews KRW, Calhoun KM, Lo N, Ho V. The aging of biomedical research in the United States. PLOS ONE. 2011;6(12): e29738 (6 pages). PMCID: PMC3247288
24. Sauermann H, Roach M. Science PhD career preferences: Levels, changes, and advisor encouragement. PLOS ONE. 2012;7(5): e3630. PMCID: PMC3342243
25. Sauermann H, Roach M. Not all scientists pay to be scientists: PhDs’ preferences for publishing in industrial employment. Res Policy. 2014;43(1): 32–47.
26. Stephan PE, Sumell AJ, Black GC, Adams JD. Doctoral education and economic development: The flow of new Ph.D.s to industry. Econ Dev Q. 2004;18(2): 151–167.
27. The disposable academic. The Economist. 2010 Dec 18;397(8713), p. 142-145.
28. Wendler C, Bridgeman B, Markle R, Cline F, Bell N, McAllister P, et al. Pathways through graduate school and into careers. Princeton: Educational Testing Service; 2012.
29. Fox MF, Stephan PE. Careers of young scientists: Preferences, prospects and realities by gender and field. Soc Stud Sci. 2001;31(1): 109-122.
30. Kemp MW, Newnham JP, Chapman E. The biomedical doctorate in the contemporary university: Education or training and why it matters. High Educ. 2012;63(5): 631–644.
31. Välimaa J. The changing nature of academic employment in Finnish higher education. In: Enders J, editor. Academic staff in Europe Westport: Greenwood Press; 2001. pp. 67–89.
32. Austin AE. Preparing the next generation of faculty: Graduate school as socialization to the academic career. J High Educ. 2002;73(1): 94–122.
33. Mason MA, Goulden M, Frasch K. Why graduate students reject the fast track. Academe. 2009;95: 11-16.
34. Gibbs KD, Griffin KA. What do I want to be with my PhD? The roles of personal values and structural dynamics in shaping the career interests of recent biomedical science PhD graduates. CBE-Life Sci Educ. 2013;12(4): 711–723. PMCID: PMC3846521
35. Roach M, Sauermann H. A taste for science? PhD scientists’ academic orientation and self-selection into research careers in industry. Res Policy. 2010;39(3): 422–434.
36. Hermanowicz JC. Scientists and satisfaction. Soc Stud Sci. 2003;33(1): 45–73.
37. Stephan PE. Science and the university: Challenges for future research. CESifo Econ Stud. 2008;4(2): 313–324.
38. Scaffidi AK, Berman JE. A positive postdoctoral experience is related to quality supervision and career mentoring, collaborations, networking and a nurturing research environment. High Educ. 2011;62(6): 685–698.
39. Lane NF. Science policy tools: Time for an update. Issues Sci Technol. 2011;28(1): 31–38.
40. Teitelbaum MS. A new science degree to meet industry needs. Issues Sci Technol. 2006;23(1): 27–30.
41. Lam A, de Campos A. “Content to be sad” or “runaway apprentice”? The psychological contract and career agency of young scientists in the entrepreneurial university. Hum Relat. 2015;68: 811–841.
42. Lam A. From “ivory tower traditionalists” to “entrepreneurial scientists”? Academic scientists in fuzzy university-industry boundaries. Soc Stud Sci. 2010;40(2): 307-340.
43. Stuart TE, Ding WW. When do scientists become entrepreneurs? The social structural antecedents of commercial activity in the academic life sciences. Am J Sociol. 2006;112(1): 97-144.
44. Coppola BP. Advancing STEM teaching and learning with research teams. New Directions for Teaching & Learning. 2009;(117): 33–44.
45. Yin RK. Case study research: Design and methods (Rev. ed.). Newbury Park: Sage Publications; 1989.
46. Boblin, SL, Ireland S, Kirkpatrick H, Robertson K. Using Stake’s qualitative case study approach to explore implementation of evidence-based practice. Qual Health Res. 2013;23(9):1267–1275.
47. Saldaña J. The coding manual for qualitative researchers. 2^nd^ ed. Los Angeles: Sage Publications; 2013.
48. Lent RW, Brown SD, Hackett G. Toward a unifying social cognitive theory of career and academic interest, choice, and performance. J Vocat Behav. 1994;45: 79-122.
49. Rubin HJ, Rubin I. Qualitative interviewing: The art of hearing data. Thousand Oaks: Sage Publications; 1995.
50. Seidman I. Interviewing as qualitative research: A guide for researchers in education and the social sciences. 4^th^ ed. New York: Teachers College Press; 2013.
51. Benderly BL. Is America’s science education gap caused by career planning fears? Pacific Standard. 2010 June. Available from: https://psmag.com/the-real-science-gap-f00edae57ba1#.31yeiru0h
52. Yewdell JW. How to succeed in science: A concise guide for young biomedical scientists: Part I: Taking the plunge. Nat Rev Mol Cell Bio. 2008;9(5): 413–416. PMCID: PMC268575
53. Jaspers K. The idea of the university. Boston: Beacon Press; 1953.
54. Merriam SB. How research produces knowledge. In: Peters JM Jarvis P, editors. Adult education. San Francisco: Jossey-Bass; 1991. pp. 42-65.
55. Ponterotto JG. Qualitative research in counseling psychology: A primer on research paradigms and philosophy of science. J Couns Psychol. 2005;52(2): 126-136.
56. Stake RE, Savolainen R. The art of case study research. Thousand Oaks: Sage Publications; 1995.
57. Yin RK. Case study research: Design and methods, 4th ed. Thousand Oaks: Sage Publications; 2009.
58. Maxwell JA. Qualitative research design: An interactive approach, 2nd ed. Thousand Oaks: Sage Publications; 2005.
59. Ravitch SM, Riggan M. Reason & rigor: How conceptual frameworks guide research. Thousand Oaks: Sage Publications; 2012.
60. Krueger RA, Casey MA. Focus groups: A practical guide for applied research. Thousand Oaks: Sage Publications; 2014.
61. Larkin M, Thompson AR. Interpretative phenomenological analysis in mental health and psychotherapy research. In: Harper D, Thompson AR, editors. Qualitative research methods in mental health and psychotherapy: A guide for students and practitioners. Hoboken: John Wiley & Sons; 2012. pp. 99-116.
62. Shaw R. QM3: Interpretative phenomenological analysis. In: Forrester M, editor. Doing qualitative research in psychology: A practical guide. Thousand Oaks: Sage Publications; 2010. pp. 177-201.
63. Smith JA, Flowers P, Larkin M. Interpretative phenomenological analysis: Theory, method and research. Thousand Oaks: Sage Publications; 2009.
